# Archaeometric perspective on the emergence of brass north of the Alps around the turn of the Era

**DOI:** 10.1038/s41598-021-04044-7

**Published:** 2022-01-10

**Authors:** Daniel Bursák, Alžběta Danielisová, Tomáš Magna, Petr Pajdla, Jitka Míková, Zuzana Rodovská, Ladislav Strnad, Jakub Trubač

**Affiliations:** 1 Institute of Archaeology of the CAS Prague v.v.i., Letenská 4, 118 01 Prague 1, Czech Republic; 2 Czech Geological Survey, Klárov 3, 118 21 Prague 1, Czech Republic; 3 Department of Archaeology and Museology, Faculty of Arts, Masaryk University, Joštova 220/13, 662 43 Brno, Czech Republic; 4 Institute of Geochemistry, Mineralogy and Mineral Resources, Faculty of Science, Charles University, Albertov 6, 128 43 Prague 2, Czech Republic

**Keywords:** Geochemistry, Computational methods, Mass spectrometry, Geochemistry, Scientific data, Metals and alloys

## Abstract

Ancient brass (*aurichalcum*) was a valued commodity in the Antiquity, notably because of its gold-like appearance. After mastering brass fabrication using the cementation procedure in the first century BC in the Mediterranean, this material became widely used by the Romans for coins, jewellery and other artefacts. Because of its visual qualities, it is believed that since this period, brass played an important role in diplomatic and economic contacts with indigenous communities, notably Celtic and Germanic tribes north of Danube and west of Rhine. To test this hypothesis, we performed for the first time the advanced statistical multivariate analysis based on chemical composition and lead isotope systematics, coupled with informed typo-chronological categorisation, of a suite of late Iron Age and Early Roman period (first century BC – first century AD) brass and other copper-alloy artefacts from the territory of Bohemia. In order to to discuss their provenance, the results were compared to known contemporary sources of material. The new results for brass artefacts from this early phase of the massive occurrence of Roman *aurichalcum* in the Barbarian territories point to the ore deposits in the western Mediterranean or the Massif Central area in Gaul, consistent with historical events. These new findings underscore the great economic and political importance of the new and rich mineral resources in the Transalpine Gaul acquired due to Caesar's military campaigns.

## Introduction

Brass is undoubtedly one of the most valued materials in Antiquity. Its high appreciation in the contemporaneous society is also underscored by the written sources, particularly in Pliny the Elder's and Cicero's works^1,2^. Since the discovery of Zn-rich alloys in the material culture of the Early Roman period, several studies have summarised its origins, the technological process of its fabrication—including the cementation—by the Romans and its importance during that period^3–7^. It appears that the widespread distribution of brass is connected with the period of the reign of Augustus and his coinage reform in 23 BC^8^.

The original brass produced by Roman workshops in the first half of the first century BC with very distinctive composition and material properties has been referred to as the *aurichalcum*^1,4,7^. Using modern analytical tools of chemical composition (XRF, EPMA, AAS, PIXE, ICP-MS), there has been some progress in identifying brass manufacture (possibly from the Roman imports) in the broader area of Europe among the artefacts dated already to the early 60s BC; however, the geological provenance of used ores remained mostly unrevealed^2,9–12^. More recently, studies systematically dealing with the Pb isotope compositions of selected materials and artefacts frequently used in the society and for constructions, such as copper^13–16^, lead^17,18^, and brass^2^, have become available. It has been noted that the provenance analyses of ancient Cu from the Iron Age and later periods might be challenging due to the complexity of the interpretations imposed by numerous and often unknown resources, widespread material mixing, recycling, depletion, or other reasons. However, even if the determination of the exact origin of the artefacts in question proves to be difficult, the provenance studies remain to be a great source of information for the understanding of contemporary socio-economic networks that often are key to understanding the historical events^18–21^.

**Figure 1 (:**
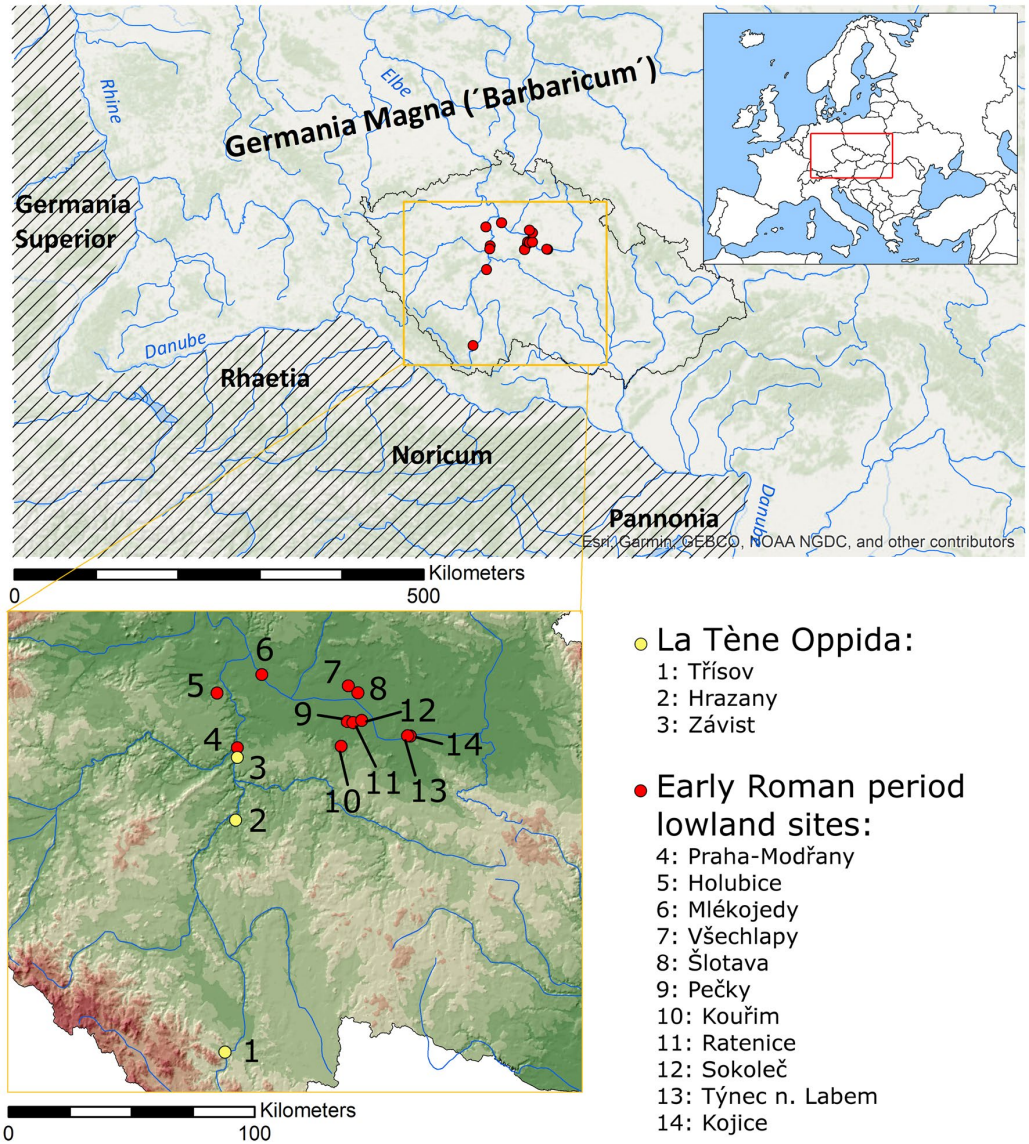
**A**) Geographical overview of Central Europe with main territorial units of the later Roman provinces (hatched areas) at the beginning of the first cent. AD; (**B**) a detailed area of interest in Bohemia with sites providing samples for this study. Background topographic map of Europe (**A**) © Esri, HERE, Garmin, Intermap, increment P Corp., GEBCO, USGS, FAO, NPS, NRCAN, GeoBase, IGN, Kadaster NL, Ordnance Survey, Esri Japan, METI, Esri China (Hong Kong), © OpenStreetMap contributors, and the GIS User Community; digital elevation model of the terrain in the (**B**) © ČÚZK, https://ags.cuzk.cz/geoprohlizec/ [ags.cuzk.cz]. Made in ArcGis software, ver.10.2.2 for Desktop (www.esri.com [esri.com]).

**Figure 2:**
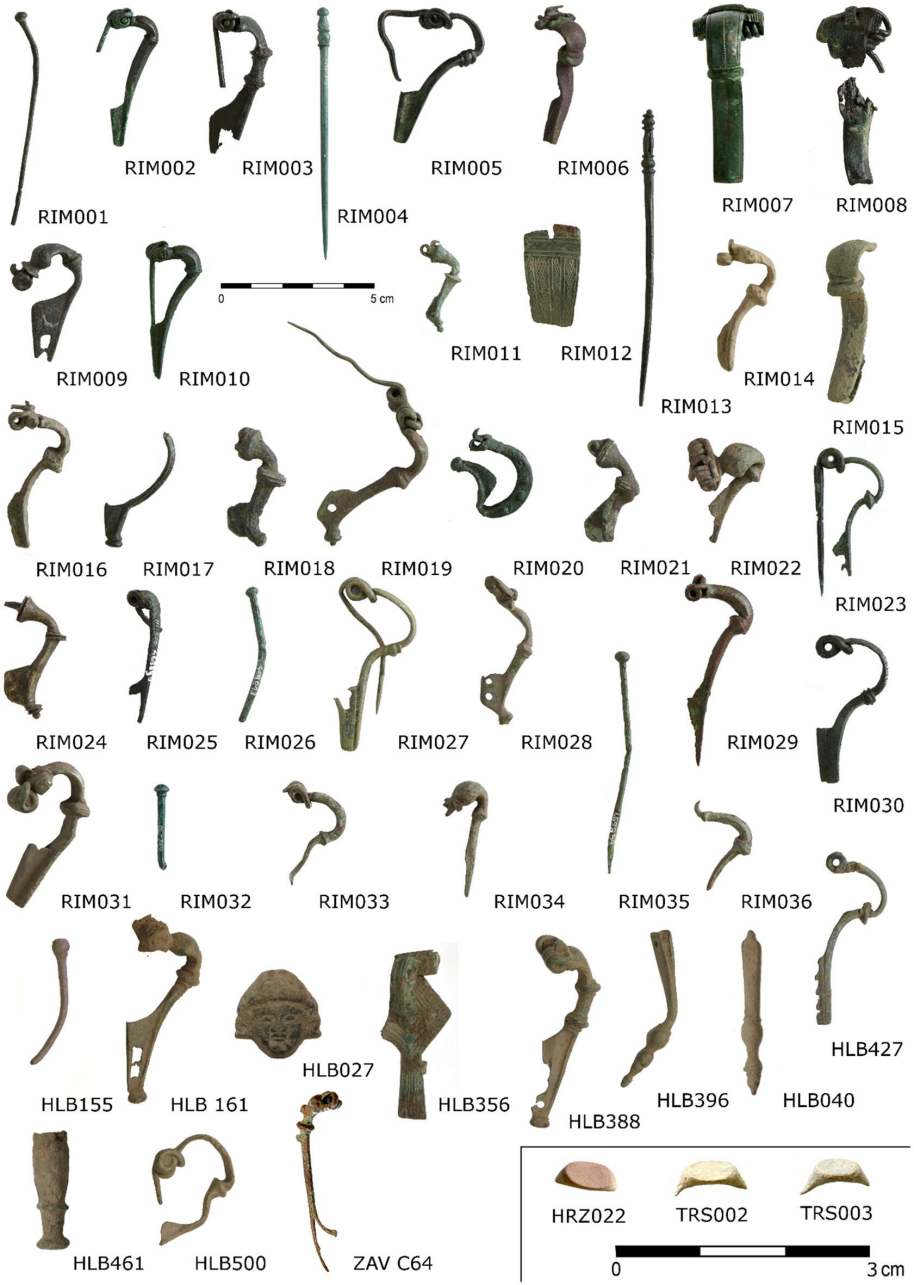
Analysed artefacts with captions corresponding to the sample description in the Tables 2, 3, 4. For further information on displayed artefacts see Suppl. Table 1.

The historical framework of this study could be briefly outlined as a period starting with the decline of the late La Tène civilisation (conventionally linked with the Celts), around the middle of the first century BC, followed by a massive migration wave(s) of the early Germanic tribes (Marcomanni, Quadi, and others) sometime between the second half of the first century BC and the second century AD^12,22^. The beginning and the course of the Julio-Claudian dynasty with a distinct intensification of the Romano–'Barbarian' contacts is the primary time frame for the majority of samples presented in this study^23–27^. In this period, jewellery from the Germanic graves in Bohemia was identified to be made of high-quality brass, approaching the chemical composition of the original *aurichalcum*^4,28,29^. A possible Roman origin of the brass coming to indigenous territories was naturally assumed when these artefacts were originally investigated^28^. Especially in the case of early Germanic brooches of the so-called 'eye type' with ca. 20 wt.% Zn, it was proposed that recycled Roman imports, primarily brass coins, were used for their fabrication^29,30^. The earliest brass artefacts in Bohemia are represented by a brass *fourrée* (counterfeit) stater from the oppidum of Stradonice^31^, brass Almgren 65 brooch from the oppidum of Závist, and several imported brass rings from the other oppida (Figs. 1, 2, Tables 2, 3, 4), all dated around the middle of the first century BC and, except for the coin, supposedly the products of Roman workshops imported to the North as exclusive jewellery pieces^12^. The striking richness and material diversity of copper-alloy artefacts in the Bohemian territory have frequently been related to the existence of the so-called Empire of Marobudus, a power structure that kept friendly relations with the expanding Roman Empire after the critical defeat of three legions in the Battle of the Teutoburg Forest in AD 9^27,32^. In that period, the Central European territory was at the intersection of the territorial interests of the expanding Roman Empire, new migration waves of the Germanic tribes from the North and the West, and the remainder of the late Celtic population. As such, this territory interconnected many cultural traditions manifested in the material culture. Those were, in particular, the costume parts (brooches, belts, pins and other personal artefacts), imported luxury items such as bronze drinking vessels, tableware, and other artefacts.

All these observations make Bohemia an exceptionally well-suited territory for studying brass production and circulation patterns compared to other 'Barbarian' territories north of the Alps and east of the Rhine River (Fig. 1). However, there still is a limited knowledge on the nature of trade with brass between the Romans and the indigenous populations (i.e. Celtic and Germanic tribes) beyond the Roman territories. The only known indication from the region north of the Alps is a hoard retrieved from the Rhine River, containing over 50 bars, some of them made of brass^30^. The massive emergence of brass artefacts north of the Alps and in Bohemia in particular, almost unknown during the preceding La Tène period, corresponds well with a significant influx of Roman imports to the Barbarian territories in the Early Roman period, i.e. between the second half of the first century BC and the first half of the first century AD^28,29,32–34^. Here, we present new compositional and Pb isotope data for the early Roman brass artefacts from the territory of Bohemia to initiate the discussion on the currently scarce archaeometric research on the emergence of early brass in Europe. The character of bronze and brass from the Early Roman period is compared with the chronologically preceding finds from the Late Iron Age^20,21,35^.

## Material

### Artefacts studied and sampling design

In total, 50 Late Iron Age and Early Roman period artefacts from the territory of Bohemia were sampled for compositional and Pb isotopic analyses (Figs. 1, 2). Most items come from metal detector prospections and the information about their original context is thus uncertain. Nonetheless, all samples can be characterised in terms of localisation, typological determination, cultural provenance and archaeological dating (Tables 1, 2). The selection of samples for further investigations was driven by identifying those artefacts among the cultural groups most likely fabricated from brass.

**Table 1:** Summary of the chronological system employed in the sample categorisation. The generally accepted chronological framework for the late La Tène^12^ and the Early Roman period^22,44,54,55^, respectively, were followed.

	La Tène period		Early roman period		
130/120–70 s BC	LT	LT D1a	-		
70 s–50/40 s BC		LT D1b	-		
from 60 s/40 s–20 s/0 BC	LT D2 / R A				
10/5 BC–AD 20/30	-		R B	R B1	R B1a
AD 20/30–40/50	-				R B1b
AD 50/70–150/160	-			R B2

**Table 2:** Description of analysed samples with their localisation, category of cultural provenance (see chapter 2.2 and Table 1), and dating. Brooches in the typological system of O. Almgren^94^ were abbreviated as A (*X*).

Lab. No	Site	GPS	Typology	Category	Dating
RIM012	Kouřim	50.0061456 N, 14.9959014E	Alesia	I	A
RIM017	Mlékojedy	50.2666303 N, 14.5442911E	Aucissa	I	A-B1
RIM020	Všechlapy	50.2261361 N, 15.0351342E	Hinge-pin brooch	I	B
HLB027	Holubice	50.1982678 N, 14.2899803E	Imported vessel	I	B
RIM005	Všechlapy	50.2261361 N, 15.0351342E	A 18_K Na	I	A-B1
RIM030	Mlékojedy	50.2666303 N, 14.5442911E	A 18	I	A-B1
RIM006	Týnec n. Labem	50.0439286 N, 15.3705692E	A 45	L	B1b
RIM008	Pečky	50.0962303 N, 15.0297508E	A 24	L	B1a
RIM011	Šlotava	50.2007908 N, 15.0897411E	Trumpet style brooch	L	B1c-B2a
RIM014	Mlékojedy	50.2666303 N, 14.5442911E	needle (costume)	L	B
RIM015	Kojice	50.0423619 N, 15.3877225E	A 45	L	B1b
RIM016	Šlotava	50.2007908 N, 15.0897411E	A 45b	L	B1b
RIM018	Sokoleč	50.1003031 N, 15.1126050E	Trumpet style brooch with Ag decoration	L	B2a
RIM021	Šlotava	50.2007908 N, 15.0897411E	Trumpet style brooch	L	B2a
RIM023	Mlékojedy	50.2666303 N, 14.5442911E	A 2a	L	B1a
RIM024	Sokoleč	50.1003031 N, 15.1126050E	Trumpet style brooch	L	B1b-B2
RIM025	Mlékojedy	50.2666303 N, 14.5442911E	Spoon brooch	L	LT D1b-D2
RIM026	Mlekojedy	50.2666303 N, 14.5442911E	needle (costume)	L	B
RIM027	Kojice	50.0423619 N, 15.3877225E	A 2a	L	B1a
RIM031	Ratenice	50.0920564 N, 15.0620225E	A 45b	L	B1b
HLB040	Holubice	50.1982678 N, 14.2899803E	Belt fitting	L	B1
HLB155	Holubice	50.1982678 N, 14.2899803E	needle (costume)	L	B
HLB396	Holubice	50.1982678 N, 14.2899803E	Belt fitting	L	B
HLB427	Holubice	50.1982678 N, 14.2899803E	A 2a	L	B1a
HLB461	Holubice	50.1982678 N, 14.2899803E	Drinking horn fitting	L	B
HLB500	Holubice	50.1982678 N, 14.2899803E	A 2a	L	B1a
RIM001	Mlékojedy	50.2666303 N, 14.5442911E	needle (costume)	L	B
RIM004	Mlékojedy	50.2666303 N, 14.5442911E	needle (costume)	L	B1
RIM007	Praha-Modřany	49.9991067 N, 14.4076225E	A 24	L	B1a
RIM013	Kojice	50.0423619 N, 15.3877225E	A 49?	L	B1b
RIM032	Mlékojedy	50.2666303 N, 14.5442911E	needle (costume)	L	B1
RIM033	Šlotava	50.2007908 N, 15.0897411E	A 2	L	B1a
RIM035	Mlékojedy	50.2666303 N, 14.5442911E	needle (costume)	L	B1
HRZ022	Hrazany	49.7343239 N, 14.4015322E	Ring	LT-Brass	LT D
TRS 002	Třísov	48.8869861 N, 14.3518881E	Ring	LT-Brass	LT D
TRS 003	Třísov	48.8869861 N, 14.3518881E	Ring	LT-Brass	LT D
ZAV C64	Závist	49.9631869 N, 14.4087258E	A 65	LT-Brass	LT D1b
RIM009	Šlotava	50.2007908 N, 15.0897411E	A 67a	N	B1a
HLB161	Holubice	50.1982678 N, 14.2899803E	A 67 -67/68	N	B1b
HLB388	Holubice	50.1982678 N, 14.2899803E	A 236a	N	B1a
RIM003	Týnec n. Labem	50.0439286 N, 15.3705692E	A 236	N	B1
RIM019	Ratenice	50.0920564 N, 15.0620225E	A 68	N	B1b-B2a
RIM022	Šlotava	50.2007908 N, 15.0897411E	A 67	N	B1
RIM028	Ratenice	50.0920564 N, 15.0620225E	A 68?	N	B1b-B2a
RIM034	Šlotava	50.2007908 N, 15.0897411E	A 67	N	B1
RIM002	Praha-Modřany	49.9991067 N, 14.4076225E	A 19aI	W	B1a
RIM029	Týnec n. Labem	50.0439286 N, 15.3705692E	A 19	W	B1
HLB356	Holubice	50.1982678 N, 14.2899803E	Feugère 19d	W	B1
RIM010	Praha-Modřany	49.9991067 N, 14.4076225E	A 19aI	W	B1a
RIM036	Šlotava	50.2007908 N, 15.0897411E	A 19	W	B1

### Categorisation of samples

A rich typological diversity of the finds led to a robust scholarly tradition in the past, which was aimed to results in a thorough typo-chronological evaluation with a great effort put into the detailed mapping, sequencing and cataloguing of the finds^36–46^. A vital research premise was to establish the general typo-chronological groups of personal jewellery and other artefacts, usually described as the 'Western tradition' (i.e. Gallic and Rhenish), the 'Danube tradition' (or Norico-Pannonian, Rhaetian), or in a more general sense the 'Roman-provincial tradition'. Such categorisation represents a valuable methodological tool for working with archaeometric data because it can be used as independent evidence.

The following cultural and chronological groups of brass artefacts from this study were defined for the correlation with the chemical analysis:

- ***La Tène ('LT***-**Brass'):** artefacts made in the late La Tène tradition of metallurgical production, usually carried out at the oppida or other major agglomerations (mainly in the Middle Danube area). Their chronological assessment and interpretation are based on a recent study of the late Iron Age in Bohemia^12^. This group contains four brass artefacts: a late Almgren 65 type brooch and three imported brass rings of the Roman provenance (Fig. 2, Table 2).
- ***Local ***('**L'**): artefacts generally supposed to be fabricated in the early 'Barbarian' metallurgical tradition. This material culture commonly is associated with the Germanic tribes appearing in the Bohemian territory from the second half of the first century BC. This group mostly contains personal artefacts, especially eye brooches of the Almgren 45–49 type^37,47,48^.
- ***Import ('I'):*** finds not of the 'Barbarian' provenance; they usually are considered to be the diplomatic gifts brought to the Bohemian territory by trade, exchange or as booty from the territories controlled by the Romans^32,49–51^. Because several different typological groups are included in this broad category, further subdivisions were needed, such as 'Noric' and 'Western'. Also, more ambivalent types in terms of the place of origin were included, such as Almgren 18 brooches, which sometimes are interpreted as being produced locally. Bohemian finds appear to be exogenous and ultimately connected with the populations that occupied the territory from the second half of the first century BC^52^.
- ***Noric*** ('N'): artefacts made in the cultural tradition of the territories around the Middle Danube zone and the Eastern Alps, including the parts of the so-called 'Norico-Pannonian costume' and finds mainly occurring in the territory of ancient Rhaetia (roughly the Alpine zone of today's Austria). Brooches and other jewellery are thought to represent the continuity from the preceding late La Tène costume tradition^36,40,43,53^.
- ***Western*** ('W'): this category has been used as a label for Alesia, Almgren 19 (including its subvariants), Almgren 15, and the so-called Gallic brooches of the types Feugère 13b, 19b and 19d. These artefacts mainly occurred around the Middle Rhine area or in eastern Gaul territory. To avoid ambiguity in cultural determination, some types, such as the Aucissa and Almgren 18, were categorised simply as ***Import*** ('I'), indicating their non-Germanic provenance.

## Results

### Chemical composition

Chemical composition of the analysed samples is summarized in Table 3. For the comparison with data published elsewhere, all compositional data used in this work are presented in the form of the analytical totals normalised to 100 wt.%.

**Table 3:** Chemical composition in wt.% of the analysed samples.

Lab. No	Cu	Fe	Co	Ni	Zn	As	Ag	Sn	Sb	Pb	Au	Cr	Mn	Pd	Mo	Ga	SUM wt.%
RIM012	85.1	bdl	0.008	bdl	18.34	bdl	1.129	0.17	0.171	2.868	0.017	bdl	bdl	0.014	0.003	0.000	100
RIM017	80.4	bdl	0.003	0.014	22.39	bdl	1.308	0.05	0.211	0.424	0.010	bdl	bdl	0.010	0.002	0.001	100
RIM020	80.3	bdl	0.002	bdl	1.73	bdl	0.437	6.56	0.010	0.216	0.041	bdl	bdl	0.010	0.002	0.001	100
HLB027	94.8	0.140	0.001	0.004	14.17	bdl	0.002	1.80	0.000	5.049	0.010	bdl	bdl	0.001	0.000	bdl	100
RIM005	88.1	0.000	0.002	0.071	3.21	bdl	0.634	4.10	0.206	0.879	0.003	bdl	bdl	0.000	0.000	bdl	100
RIM030	84.5	0.120	0.002	0.039	5.22	bdl	0.542	5.43	0.127	0.883	0.001	bdl	bdl	0.000	0.000	0.001	100
RIM006	76.8	bdl	0.001	bdl	16.56	bdl	0.470	1.17	0.059	0.182	0.023	bdl	bdl	0.003	0.000	0.001	100
RIM008	70.2	bdl	0.003	0.218	19.92	bdl	20.835	0.10	0.213	0.136	0.057	bdl	bdl	0.012	0.003	0.001	100
RIM011	90.5	bdl	0.005	bdl	20.17	bdl	1.192	0.01	0.090	0.891	0.019	bdl	bdl	0.015	0.004	bdl	100
RIM014	81.1	bdl	0.002	bdl	15.52	bdl	0.791	2.15	0.010	0.336	0.007	bdl	bdl	0.007	0.001	0.001	100
RIM015	78.2	bdl	0.002	bdl	3.83	bdl	1.370	3.61	0.129	0.255	0.010	bdl	bdl	0.009	0.002	bdl	100
RIM016	79.3	bdl	0.001	bdl	18.78	bdl	0.295	0.23	0.007	0.167	0.007	bdl	bdl	0.007	0.001	0.001	100
RIM018	91.0	bdl	0.003	bdl	15.01	bdl	1.003	1.33	0.079	0.386	0.028	bdl	bdl	0.008	0.001	bdl	100
RIM021	81.9	bdl	0.004	bdl	19.14	bdl	1.438	0.48	0.083	0.215	0.025	bdl	bdl	0.012	0.002	bdl	100
RIM023	79.4	bdl	0.005	bdl	8.88	bdl	0.600	5.32	0.018	0.353	0.021	bdl	bdl	0.011	0.002	bdl	100
RIM024	84.2	bdl	0.003	bdl	18.03	bdl	0.957	0.34	0.177	0.445	0.016	bdl	bdl	0.009	0.002	bdl	100
RIM025	79.6	bdl	0.002	bdl	bdl	bdl	0.876	10.11	0.007	1.144	0.011	bdl	bdl	0.006	0.001	bdl	100
RIM026	88.4	bdl	0.006	bdl	bdl	bdl	1.041	13.45	0.108	0.321	0.015	bdl	bdl	0.008	0.001	bdl	100
RIM027	85.6	bdl	0.001	0.015	20.06	bdl	0.643	0.03	0.297	0.014	0.013	bdl	bdl	0.003	0.000	bdl	100
RIM031	77.9	bdl	0.002	bdl	20.12	bdl	0.786	0.60	0.059	0.470	0.010	bdl	bdl	0.007	0.001	bdl	100
HLB040	85.1	0.890	0.004	0.003	0.01	bdl	0.004	0.03	0.043	0.189	0.014	bdl	bdl	0.001	0.001	0.001	100
HLB155	81.9	0.350	0.000	0.006	12.08	bdl	0.002	1.67	0.003	0.245	0.006	bdl	bdl	0.001	0.000	0.001	100
HLB396	83.9	0.410	0.001	0.003	17.38	bdl	0.004	0.09	0.111	0.336	0.018	bdl	bdl	0.001	0.000	bdl	100
HLB427	92.3	0.310	0.001	0.044	16.99	bdl	0.003	0.39	0.001	0.241	0.083	bdl	bdl	0.002	0.001	bdl	100
HLB461	89.9	0.370	0.007	0.053	22.13	bdl	0.005	0.13	0.001	9.512	0.019	bdl	bdl	0.001	0.000	bdl	100
HLB500	87.6	0.480	0.000	0.012	10.94	bdl	0.004	0.45	0.004	0.302	0.012	bdl	bdl	0.001	0.000	bdl	100
RIM001	77.7	0.130	0.001	0.060	14.34	bdl	0.321	0.87	0.090	0.388	0.001	bdl	bdl	bdl	0.000	0.001	100
RIM004	91.4	0.001	0.001	0.055	6.88	bdl	0.279	0.14	0.036	0.056	0.001	bdl	bdl	bdl	0.000	bdl	100
RIM007	78.4	0.070	0.000	0.020	0.04	bdl	2.484	0.09	0.045	0.483	0.012	bdl	bdl	bdl	0.000	0.001	100
RIM013	79.0	0.050	0.000	0.015	11.51	bdl	0.144	0.04	0.028	0.339	0.001	bdl	bdl	bdl	0.000	bdl	100
RIM032	90.4	bdl	0.003	0.039	20.82	bdl	0.082	2.00	0.049	0.047	0.001	bdl	bdl	bdl	0.000	bdl	100
RIM033	77.6	bdl	0.000	0.020	20.47	bdl	0.338	0.86	0.010	0.245	0.000	bdl	bdl	bdl	0.000	bdl	100
RIM035	79.0	bdl	0.001	0.051	15.45	bdl	0.303	1.40	0.047	0.698	0.002	bdl	bdl	bdl	0.000	0.000	100
HRZ022	76.9	bdl	0.001	0.091	bdl	0.000	0.042	8.21	0.038	0.084	0.000	0.002	0.000	bdl	bdl	bdl	100
TRS 002	75.9	0.060	0.001	0.084	2.04	0.013	0.057	8.09	0.063	0.113	0.000	0.013	0.002	bdl	bdl	bdl	100
TRS 003	78.4	0.350	0.008	0.060	17.86	0.041	0.027	0.62	0.031	0.570	0.000	0.007	0.000	bdl	bdl	bdl	100
ZAV C64	74.9	0.990	0.001	0.082	21.78	0.000	0.046	0.04	0.027	0.286	0.000	0.013	0.002	bdl	bdl	bdl	100
RIM009	82.2	bdl	0.005	bdl	20.33	bdl	1.232	0.10	0.024	0.543	0.029	bdl	bdl	0.015	0.004	0.001	100
HLB161	81.4	0.90	0.001	0.003	21.70	bdl	0.003	1.21	0.013	0.231	0.020	bdl	bdl	0.002	0.000	0.001	100
HLB388	87.1	0.100	0.001	0.024	21.12	bdl	0.005	0.15	0.005	0.424	0.012	bdl	bdl	0.001	0.000	0.001	100
RIM003	80.8	0.950	0.003	0.050	23.23	bdl	0.656	0.03	0.034	0.616	0.003	bdl	bdl	bdl	0.000	0.001	100
RIM019	76.4	0.130	0.001	0.012	9.45	bdl	0.331	4.31	0.130	0.137	0.002	bdl	bdl	bdl	0.000	0.000	100
RIM022	76.9	0.490	0.000	0.018	0.03	bdl	1.222	9.32	0.011	0.127	0.001	bdl	bdl	bdl	0.000	0.002	100
RIM028	76.2	0.030	0.000	0.007	21.10	bdl	0.262	0.64	0.085	0.136	0.002	bdl	bdl	bdl	0.000	0.000	100
RIM034	75.8	0.110	0.000	0.020	22.90	bdl	0.517	0.27	0.010	0.363	0.000	bdl	bdl	bdl	0.000	0.001	100
RIM002	80.4	bdl	0.003	bdl	18.97	bdl	0.925	0.89	0.010	0.090	0.012	bdl	bdl	0.011	0.002	0.000	100
RIM029	79.2	bdl	0.002	bdl	21.66	bdl	0.434	0.25	0.008	0.234	0.009	bdl	bdl	0.006	0.001	0.001	100
HLB356	77.2	0.270	0.000	0.026	21.37	bdl	0.002	2.28	0.013	0.187	0.020	bdl	bdl	0.001	0.001	0.000	100
RIM010	77.2	bdl	0.000	0.055	19.01	bdl	0.496	1.55	0.162	0.214	0.001	bdl	bdl	bdl	0.000	0.002	100
RIM036	77.1	0.100	0.000	0.017	20.69	bdl	0.048	2.91	0.012	0.768	0.000	bdl	bdl	bdl	0.000	0.001	100

**Figure 3:**
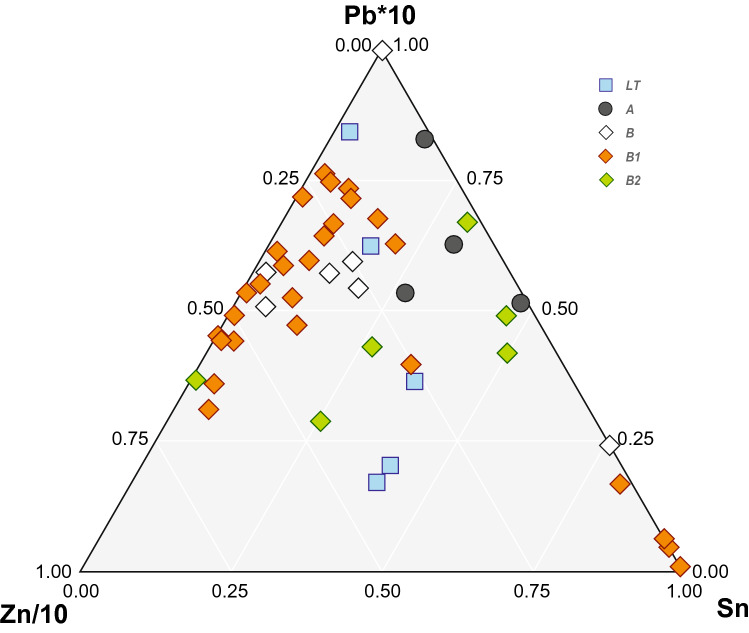
Ternary Pb–Zn-Sn plot for the Bohemian brass samples from this study categorised according to their dating. Zinc content is divided by a factor of ten, whereas the Pb content is multiplied by factor of ten. For categorisation according to the cultural groups see Suppl. Figure 1.

Majority of analysed samples is represented by Cu or Cu-based alloys with high Zn contents (Suppl. Figure 2). The visible divide within the group of brass artefacts occurs at 15 wt.% Zn (Suppl. Figure 2a). Seventeen of the 50 samples in Table 3 belong to J. Riederer’s category of Roman brass with very high Zn content (above 20 wt. %^56^) and the absence of Pb and Sn. However, four of these samples also contained >1 wt.% Sn. It is worth mentioning that such an elevated Sn content in otherwise “pure” Cu–Zn alloys is present in samples from the late Iron Age and marks a recognisable difference from the later Roman Cu–Zn alloy. Ten samples belong to Riederer’s category of Roman brass with high Zn content (10–20 wt. %^56^); 9 samples represent the category of Roman Sn -brass with high Zn content (Zn 10–20 wt. %, Sn 1–10 wt. %^56^). Samples with Zn content below approx. 16 wt. % tend to have slightly increased Sn content, up to 2.1 wt. %. Four samples were detected to be made of Sn bronze without the addition of Zn. All belong to the 'L' category, while only two of them are Sn-rich bronzes (Sn > 10 wt.%.). Generally, variations in main alloying components—Pb, Zn and Sn—(Fig. 3) are most likely compatible with different chronology. Very high Zn contents (median at 17 wt.%) on the one hand and very low Sn and Pb concentrations on the other are typical for the phase R B1 (10 BC-AD 50). There is a slight tendency towards more consistent and higher Zn contents among samples towards the end of this phase (ca. AD 30–50).

Brass with significantly lower Zn contents (2–9 wt. %) was detected in categories of imports ('I') from the beginning of the Early Roman period (second half of the first century BC). These artefacts also have similiar contents of Zn and Sn. The second category with a similar position in the composition plot (Fig. 3; Suppl. Figure 1) is partly represented by samples of the local ('L') origin that usually are dated around the middle of the first century AD (phase R B2). There are also two leaded Cu alloys: (i) a drinking horn fitting, and (ii) a fragment of a handle of the Roman imported vessel (bronze decoration with a human mask). A single case of a brooch (sample RIM008) with an exceptionally high Ag content of 20.8 wt. % was also observed.

**Figure 4:**
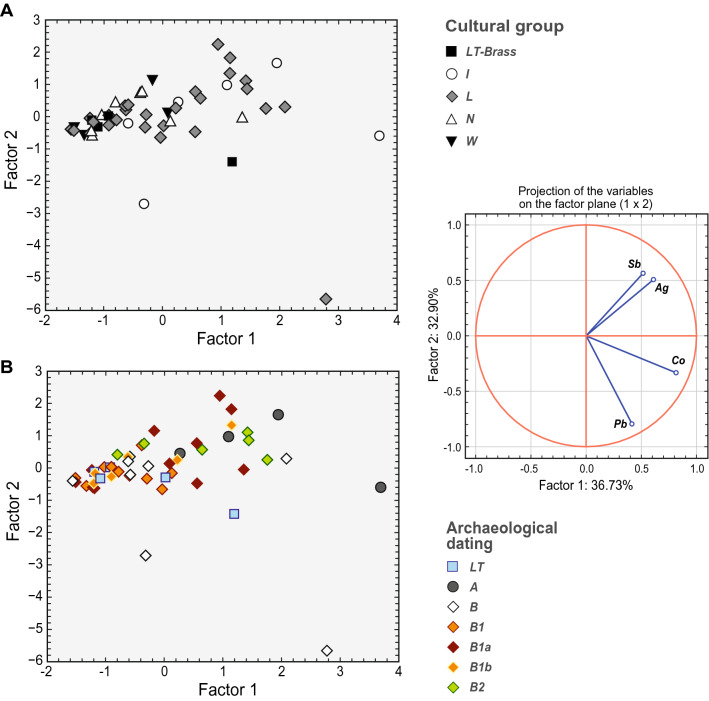
Plot with results of principal component analysis based on minor/trace elements (Pb, Co, Sb, Ag) for the Bohemian brass artefacts from this study. Factor 1 versus Factor 2 is categorised according to the cultural groups (**A**) and dating (**B**). The inset panel shows variables factor map.

Principal component analysis (PCA) was performed for a more detailed statistical evaluation of the chemical composition of samples from this study. The choice of minor/trace elements (Pb, Co, Sb, Ag) has been made considering their symptomatic value for provenance studies^57,58^. The resulting factor scores are plotted in Fig. 4. The most extensive dispersion is observed in the category of local items ('L'), followed by the imported items ('I'), reflecting significant variations in the general composition of the used alloys (Fig. 3). Most samples in the 'N' category tend to have higher Ag contents (0.26 – 1.2 wt. %) which may show consistency with their supposed Alpine origin (see below). A slightly negative correlation between Co and Ag was observed; however, there does not appear to be a clear correlation with the typo-chronological categorisation of samples (Fig. 4).

### Lead isotope systematics

**Figure 5:**
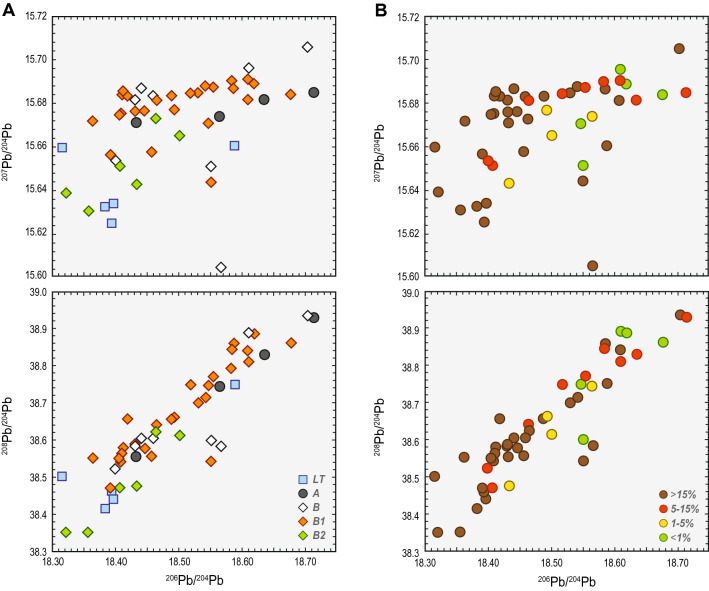
The Pb isotope systematics of the Bohemian brass artefacts from this study categorised according to the archaeological dating (**A**) and Zn contents (**B**). For categorisation according to the cultural groups see Fig. 6.

**Figure 6:**
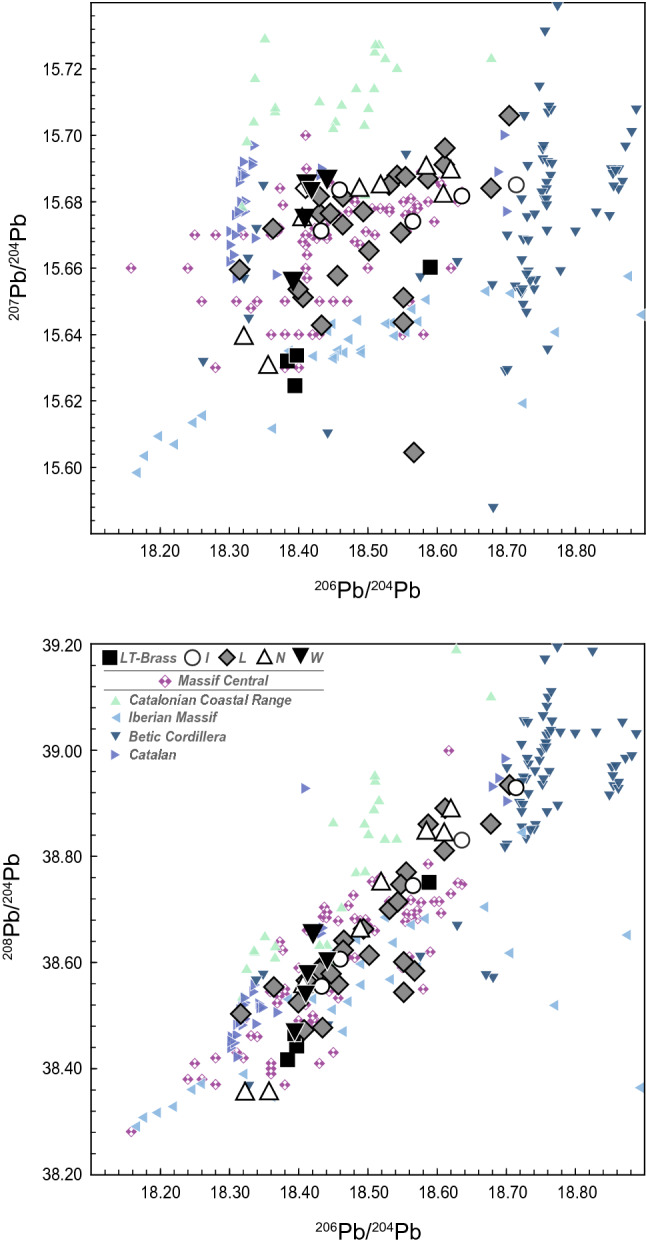
The Pb isotope systematics of ore sources from the northwest Mediterranean zone ^2,16,20,21,35,59–61^ and the Bohemian brass (this study) artefacts categorised according to the cultural groups.

Lead isotope analysis shows similar results as the PCA (Fig. 5). The most significant variability is observed in the category of local items ('L'), followed by a slightly more homogeneous Norican ('N') group and the imports ('I'). Samples in the 'Western' category ('W') form the tightest cluster, which is also coherent chronologically (phase R B1; Fig. 5a). Their linear trend and its spatial overlap are most similar with the Pb isotope systematics of the ores from the Massif Central (Fig. 6). Almost all samples from the 'LT-brass' category are mutually close and show a significant consistency with the ore deposits in the western Mediterranean (i.e. Iberia) and the Massif Central in France. Sample TRS 003 is offset from the rest of the suite but still plots in the Pb isotope space of the Spanish or French deposits.

All brass artefacts in the 'I' category (imports) are from the phase R A, and all of these samples show a tendency towards more radiogenic ^206^Pb/^204^Pb ratios. A tendency towards less radiogenic ^207^Pb/^204^Pb and ^206^Pb/^204^Pb ratios is observed for items from the chronologically youngest period (R B2). A costume pin sample RIM 001 made of brass with high Zn content (> 15 wt.%) represents an 'outlier' with the lowest ^207^Pb/^204^Pb. A clear tendency of samples with low Zn content (< 5 wt.%), dated either in the earliest or in the latest phase (R A or R B2), towards less radiogenic ^207^Pb/^204^Pb values is apparent (Fig. 5b). The samples from the phase R B1 with high Zn contents appear to be dispersed around ^206^Pb/^204^Pb value of 15.68 and are relatively homogeneous. Lead-rich (~ 5 wt.%) imported vessel has the same Pb isotope systematics as the high-Zn low-Pb items from the phase R B1. A drinking horn fitting with the highest Pb content of 9.5 wt. % plots separately with ^207^Pb/^204^Pb and ^206^Pb/^204^Pb ratios of 18.55 and 15.65, respectively.

## Discussion

The original *aurichalcum,* i.e. the brass produced in Rome, must have contained at least 22–28 wt.% Zn^4^. However, the content of Zn in aurichalcum started to decrease already in the first century AD and later produced *aurichalcum* further continued to lose its original qualities^62^. It was shown that alloy with the Zn content between 10 and 15 wt.%, that could be produced via a simple dilution process of equal quantities of bronze and the *aurichalcum,* already yielded its typical golden colour that was in demand among the indigenous communities^1,4,63^.

Those were especially the visual qualities of brass that were favoured for producing costume parts such as brooches, rings, pins and belts made both in Roman and Barbarian cultural environments. However, geochemical data for metal finds from the Barbaricum are still sparse, particularly those of the local (i.e. Germanic) provenance. In parallel to such compositional evolution on the Roman side, lower Zn contents in Barbarian artefacts measured here can be attributed to local mixing and recycling of the imported objects. In addition, a repeating pattern of small contents of Sn was observed randomly occurring in high Zn-brass artefacts throughout the entire period except for the earliest and youngest samples (Table 3).

In order to reveal further details about the manufacturing of brass in the indigenous territories, the assemblage analysed in this study was compared with chronologically and typologically compatible data from published reports. Given the overall purity of the analysed alloys in terms of chemical composition, we can exclude mixing of brass with Sn bronzes and leaded brass/bronze alloys known from the Roman Cu-alloy production for most of our samples with high Zn content^56^. A scenario of a ‘melting pot of all Cu-alloys’ may thus be definitively excluded. However, mixing of brass with a close chemical fingerprint is still plausible but, in such case, the Zn content would be lowered^4^. In case of artefacts with significantly lower Zn contents (2–9 wt. %), we may assume (i) technological experimenting given the inexperience with new material in the earliest Roman period (R A; Fig. 3; Suppl. Figure 2b), and/or (ii) repeated recycling of various Cu-alloys, including brass, tin-brass, leaded brass etc. The later process leads to a gradual depleting of alloying components with a lower evaporation point, such as Zn and Sn. In our group of samples this seem to occur more towards the end-period (R B2; Fig. 3; Suppl. Figure 2b) and correspond with the contemporary findings from elsewhere within the territories with imported Roman brass^64^. This chemical pattern can also indicate gradual lowering of the quality of the used materials that was also reported in the case of Roman coins from the same period^65^.

### Comparison with contemporary assemblages

**Figure 7:**
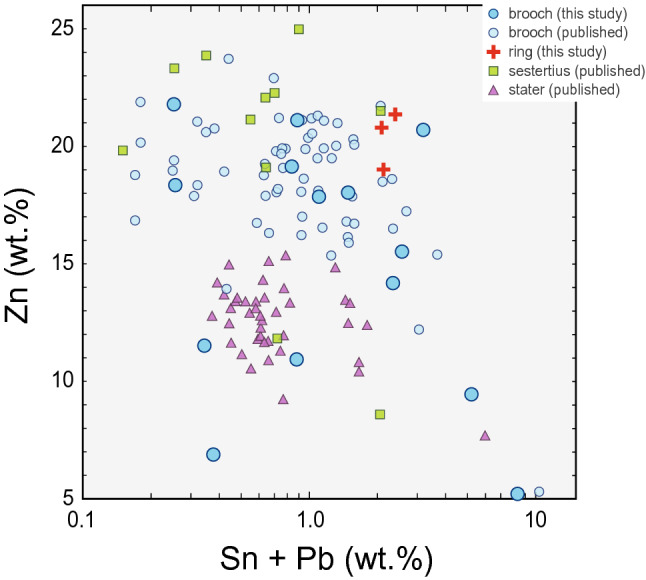
Plot of Zn verus Sn + Pb contents (in wt. %) of the earliest brass artefacts from the first century BC. For categorisation according to dating see Suppl. Figure 3 Sources:^9,10,66^ + this study.

The comparative dataset of the chemical compositions includes early Roman finds from Bohemia (NAA method^28^), and Cambodunum (AAS method^66^), both analysed in the 1990s, brass brooches from the territory of Slovenia (PIXE method^9,10^), and brass staters from Gaul (FNAA^67^). Due to the currently leading hypothesis about the Roman brass used for the fabrication of the costume parts in the Barbaricum, chemical data from the brass coinage^65^ were used for a more detailed comparison. The latest data for the Roman brass coinage were obtained by PIXE^68^ and, unfortunately, do not provide sufficiently accurate results for the comparison. When comparing the earliest brass artefacts from the first century BC, the main alloying components (Zn, Sn, Pb) reveal a cluster of late Iron Age brass staters of VERCA and Vercingetorix CAS series because of their lower mean Zn contents (ca. 12.2 wt.%; Fig. 7). The difference between coins and other brass artefacts of the assemblages mentioned above is probably due to chronology because the 'pure' brass, with high Zn contents (> 15 wt.%), and low contents of Sn and Pb (sum below 0.5 wt.%) appeared no earlier than around the Augustan period. Besides, there is evidence of Zn-rich brass in the second half of the first century BC (phase R A; Suppl. Figure 2, 3).

A second PCA with only selected trace elements only (Co, Ni, Sb, Ag) was carried out including the comparative datasets. To avoid inconsistency, the dataset was reduced solely to brooches. This step further enhanced the chronological compatibility among the typological groups of the artefacts. Also, in the archaeological categorisation of the groups, several trends were revealed (Suppl. Figure 4a). Similarly to 'N' samples from Bohemia, also Norican brooches tend to contain more Ag, which is paralleled by higher contents of Sb, thus indicating fahlore copper^69,70^ used for their fabrication. The most significant variability in the trace element composition was observed for the local items ('L'). Chronologically speaking, a notable heterogeneity in the chemical composition may be observed in the phase R B1a compared to the following phase R B1b (Suppl. Figure 4b). A specific group of the artefacts of the Norican tradition from the phase R B2 forms a tight cluster in both plots. These findings indicate rather heterogeneous supply patterns of brass in the beginnings of the trade contacts between the early Empire and the Germanic communities, compared to the late Republic and Celtic agglomerations of the second and first century BC on the one hand and late first century AD on the other.

When these results are compared with the Roman copper AES coinage, a weak correlation between the part of the Bohemian samples with elevated Sb and Ag levels (Sb 0.01–0.2 wt. %; Ag 0.001–1.5 wt. %.) and the AES coinage elemental pattern group III (EPG III), characterised also by increased levels of Sb (0.02–0.1 wt. %) and Ag (0.03–0.7 wt. %), is apparent^13^. Similar Sb- and Ag-rich copper AES coinage is also specific for the Lyon altar series I (LAS I) AES coinage^71^.

**Figure 8:**
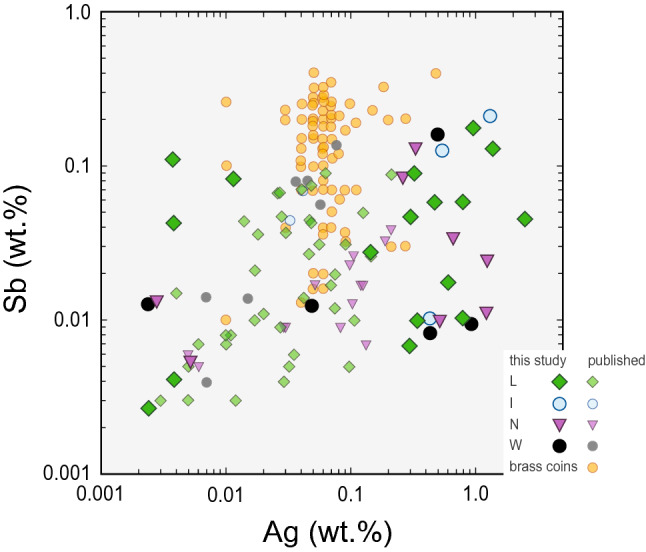
Plot of Sb versus Ag contents (in wt. %) of brass artefacts with > 5 wt.% Zn from Bohemia and Cambodunum with the Roman brass coinage categorised according to the cultural and chronological groups. Sources: this study;^65,66^.

The comparison of brass artefacts with > 5 wt.% Zn from Bohemia and Cambodunum^66^ with the Roman brass coinage^65^ is based on the systematics of Sb and Ag (Fig. 8). It clearly shows the incompatibility between the local brass artefacts and the Roman *sestertii*. Due to the different analytical approaches to obtain the compositional data, the results must be treated with caution. Also, a significant variability of the Roman metal supply for the coin production [cf.^13^] should be considered. Therefore, for successful future provenance studies, a targeted archaeometric analysis of the Roman coins is vital.

A brooch with an unusually high Ag content (20.8 wt. %) is, generally speaking, uncommon—even in the context of the broad spectrum of Early Roman finds from Central Europe^29,66^. Nonetheless, there are artefacts known from the contemporary cemeteries to had been manufactured from pure Ag, and particularly this type of brooch (Almgren 24) is the one most frequently fabricated artefacts from precious metals^48,72^. Considering the technologically advanced metallurgy – both Roman and Barbarian – unintentional contamination caused by the accidental use of Ag-rich ore is considered rather implausible. Local manufacturing of Ag-rich brooches by deliberate alloying with Ag thus remains a possible explanation.

### Provenance analysis – mineral exploitation and raw resources in the Early Roman period

**Figure 9:**
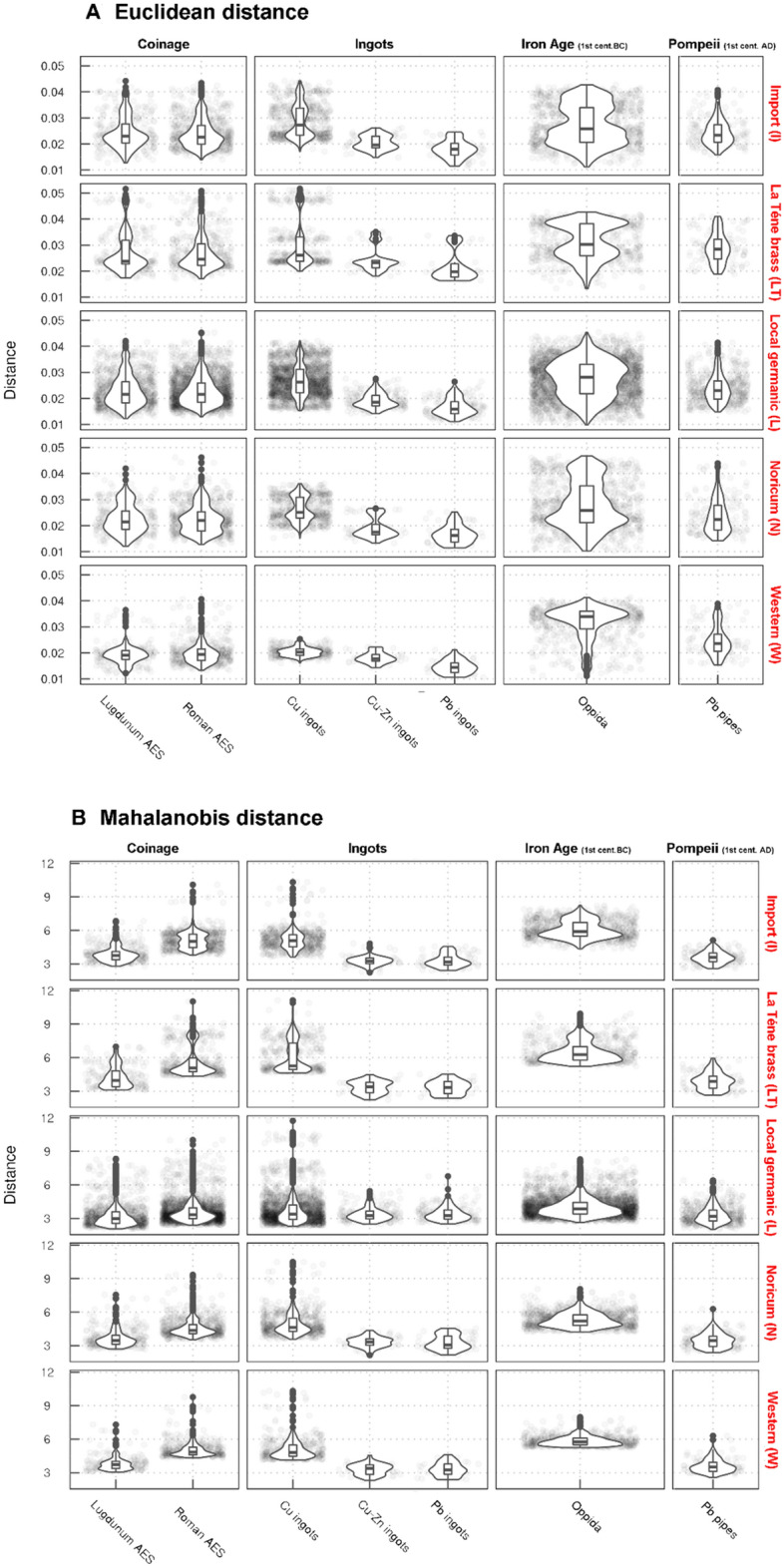
Euclidean (ED) and Mahalanobis (MD) distances between Pb isotope ratios of various sets of the analysed artefacts from the Early Roman period (red labels) and the comparative datasets of bronze and brass artefacts (coinage, ingots, Iron Age objects from the oppida, Pompeii objects; for references, see main text). The graph shows the density distributions (‘density violin plots’) according to their distance. Unlike ED, the MD distributions also take into consideration the shape of the data clouds and their trends. The closer to zero the higher the probability of the analytical match. Sources: see description in the main text + this study.

Lead isotope analysis has become a conventional method for tracing the archaeological artefacts containing Pb to their possible geological origins, i.e. the ores the artefacts were fabricated from^73,74^. The provenance analysis testing the consistency between the samples and the known ore deposits was carried out using a combination of the conventional biplots and the Euclidean (ED) and Mahalanobis (MD) distance algorithms^21^. The same approach was then applied to compare the contemporary bronze and brass assemblages of various cultural backgrounds. The ED algorithm has initially been suggested by Stos^75^ as a simple metric to compare how far the point distributions are from one another in a multivariate space defined by individual Pb isotope signals. While the ED algorithm is currently widely used, it is advised to be complemented with MD in which the effects of the shape, scale and trend of the distribution of the data are accounted for^76^. Therefore, the metric can measure the distance from a data point to distribution in the multivariate space and can account for the distance of points as well as for the linear trends in the data and distributions of the data clouds. Plots derived using such an approach (Fig. 9; Suppl. Figure 5) are then used to predict the allocation of the analysed brass artefacts from Bohemia in the comparative datasets. Due to considerable overlaps in the data distributions of different ore deposits, we note that the predictive value of ED + MD can vary among distinctive sources.

Because 92% (n = 50) of our samples have a low Pb content (< 1 wt.%), we do not consider the sampled alloys to have been deliberately leaded^56,64,77^. The Pb isotope systematics thus appear to reflect the intrinsic mineral-derived lead than can be used for reliable prediction of their provenance. The best level of consistency for most of our samples is observed with polymetallic deposits from the Massif Central (Fig. 6; Suppl. Figures 5, 6). These results were verified by both the ED and MD algorithms; however, the outliers in the Massif Central ore dataset provided a less pronounced consistency than the standard biplot. There is also a possibility of mixing the sources from various deposits, namely the Mediterranean (Iberia, Sardinia, Macedonia, or Attica) or the Alpine (namely the south-eastern Alps and the Inn Valley; Suppl. Figures 5, 6). The Alpine signal is the most apparent in the 'N' category. British source ores did not come into consideration until AD 43 when Britain came under the Roman control and data were thus omitted for historical reasons.

Additional chemical data from the early Roman imperial Pb artefacts were included in the comparative analysis: the Augustan Pb water pipes from Pompeii [code 'Pb pipes';^78^] and Pb ingots from the shipwreck of Sainte-Maries-de-la-Mer [code 'Pb ingots';^59^]. A similar analytical match and subsequent historical interpretation favouring the Massif Central deposits were suggested for brass ingots from the Aléria shipwreck [code 'Cu–Zn Ingot'; ^2^].

Because no Pb isotope data for the artefacts of the 'Barbarian' provenance are available, only the early Roman Imperial datasets could be included in the comparative analysis. Regarding the provenance of Cu, there is an extensive corpus of comparative data from the Roman AES coinage^14,71^ and Cu ingots of the Sud-Lavezzi 2 Bonifacio wreck from the beginning of the first century AD^15^. Finally, since we aimed to reveal a possible consistency between the Germanic finds and the preceding late Iron Age artefacts, bronze artefacts mainly from the first century BC ('Oppida' set) were also included in the comparative dataset^20,21,35^ to detect potential looting of the abandoned Celtic oppida by the newly incoming Germanic populations.

The results show that the Pb isotope compositions of most of the Early Roman samples in this study are generally inconsistent with late Iron Age finds (cf. results of ED and MD, Fig. 9; Suppl. Figure 7). A subset of samples, consisting mostly of artefacts dated to the La Tène period or late first and/or second century AD with less radiogenic ^207^Pb/^204^Pb and ^206^Pb/^204^Pb ratios are closer to ore deposits in Germany (Suppl. Figure 6), and are consistent with Roman Cu coins, Cu–Zn, Pb ingots and part of the copper AES coinage from the LAS I. There appears to be a consistency with the preliminary findings of Roman copper/brass alloys from Elsfleth-Hogenkamp dated to the second – third century AD as well, but we note that the report of Merkel lacks analytical data and we cannot make any further conjectures^77^. No analytical match with the Cu bars from Sud-Lavezzi 2 Bonifacio was observed, although the Cu bars might be a very convenient and contemporary source of copper. The samples with low Zn contents (Fig. 3; Suppl. Figure 2) from the R A and R B2 phases, respectively, combined with their tendency towards less radiogenic ^207^Pb/^204^Pb ratios (Fig. 5b), are still within the range of the ores from the Massif Central. However, three out of four samples from the early phase (R A) show some proximity towards the south-eastern Iberian zone, most compatible with the late La Tène samples^20^. Sample RIM017 with a lower ^206^Pb/^204^Pb ratio contradicting this chronological explanation could equally be dated into the phase R B1 (Tables 2, 3, 4). The LAS I is consistent with a subset of the Bohemian samples in their Pb isotope ratios and Ag contents^71^. A tendency towards less radiogenic ^207^Pb/^204^Pb and ^206^Pb/^204^Pb ratios may result from the influence of the Iberian Massif (Fig. 6; Suppl. Figure 5, 6). The southern Spanish mines are thought to be most important in the organisation of the Roman Cu supply^14^, which is also evidenced by the most chronologically compatible dataset—the imperial AES copper coinage. Data from the AES coinage are partly inconsistent with Cu ingots, but the variability of Cu sources corresponds well with the suggested complexity of the Cu industry of the Roman Empire^15^. In the case of samples from the phase R B2, i.e. after 43 AD, Pb–Zn deposits from Great Britain may also come into consideration [cf.^2^]. Since data from this late phase is rather subordinate in number, further historical analysis, such as the trend comparisons with discussed ore deposits, cannot be performed.

**Table 4:** Analysed samples with values of Pb isotope ratios.

Lab. No	^206^Pb/^204^Pb	^207^Pb/^204^Pb	^208^Pb/^204^Pb	^207^Pb/^206^Pb	^208^Pb/^206^Pb	Pb group	Zn group
RIM012	18.714	15.685	38.929	0.838	2.081	> 1% < 5%	> 5% < 15%
RIM017	18.433	15.671	38.555	0.850	2.092	< 1%	> 15%
RIM020	18.441	15.687	38.606	0.850	2.094	< 1%	> 15%
HLB027	18.460	15.684	38.607	0.849	2.092	> 5%	> 15%
RIM005	18.565	15.674	38.745	0.844	2.087	< 1%	> 1% < 5%
RIM030	18.636	15.682	38.830	0.841	2.084	< 1%	> 5% < 15%
RIM006	18.431	15.676	38.589	0.850	2.094	< 1%	> 15%
RIM008	18.493	15.677	38.662	0.847	2.091	< 1%	> 1% < 5%
RIM011	18.434	15.643	38.477	0.848	2.088	< 1%	> 1% < 5%
RIM014	18.704	15.706	38.936	0.840	2.082	< 1%	> 15%
RIM015	18.542	15.688	38.715	0.846	2.088	< 1%	> 15%
RIM016	18.410	15.684	38.566	0.852	2.095	< 1%	> 15%
RIM018	18.502	15.665	38.614	0.846	2.087	< 1%	> 1% < 5%
RIM021	18.464	15.673	38.623	0.849	2.092	< 1%	> 15%
RIM023	18.364	15.672	38.554	0.853	2.100	< 1%	> 15%
RIM024	18.407	15.651	38.473	0.850	2.090	< 1%	> 5% < 15%
RIM025	18.316	15.660	38.504	0.855	2.103	> 1% < 5%	> 15%
RIM026	18.611	15.696	38.891	0.843	2.090	< 1%	< 1%
RIM027	x	x	x	x	x		
RIM031	18.447	15.677	38.579	0.850	2.092	< 1%	> 15%
HLB040	18.611	15.691	38.811	0.843	2.086	< 1%	> 5% < 15%
HLB155	18.431	15.682	38.585	0.851	2.094	< 1%	> 15%
HLB396	18.400	15.654	38.525	0.851	2.094	< 1%	> 5% < 15%
HLB427	18.555	15.688	38.771	0.845	2.090	< 1%	> 5% < 15%
HLB461	18.552	15.651	38.601	0.843	2.081	< 1%	< 1%
HLB500	18.465	15.682	38.642	0.849	2.093	< 1%	> 5% < 15%
RIM001	18.567	15.605	38.584	0.840	2.078	< 1%	> 15%
RIM004	18.548	15.671	38.748	0.845	2.090	< 1%	< 1%
RIM007	18.457	15.658	38.559	0.848	2.089	< 1%	> 15%
RIM013	18.531	15.685	38.700	0.846	2.089	< 1%	> 15%
RIM032	18.678	15.684	38.861	0.840	2.081	< 1%	< 1%
RIM033	18.587	15.687	38.861	0.844	2.091	< 1%	> 15%
RIM035	18.552	15.644	38.544	0.843	2.078	< 1%	> 15%
HRZ022	18.397	15.634	38.442	0.850	2.090	< 1%	> 15%
TRS 002	18.384	15.632	38.417	0.850	2.090	< 1%	> 15%
TRS 003	18.589	15.660	38.752	0.842	2.085	< 1%	> 15%
ZAV C64	18.394	15.625	38.464	0.849	2.091	< 1%	> 15%
RIM009	18.584	15.690	38.845	0.844	2.091	< 1%	> 5% < 15%
HLB161	18.519	15.685	38.749	0.847	2.093	< 1%	> 5% < 15%
HLB388	18.620	15.689	38.887	0.842	2.089	< 1%	< 1%
RIM003	18.609	15.682	38.842	0.842	2.088	< 1%	> 15%
RIM019	18.322	15.639	38.352	0.853	2.094	< 1%	> 15%
RIM022	18.489	15.684	38.658	0.848	2.091	< 1%	> 15%
RIM028	18.357	15.630	38.353	0.851	2.090	< 1%	> 15%
RIM034	18.406	15.675	38.554	0.851	2.095	< 1%	> 15%
RIM002	x	x	x	x	x	< 1%	> 15%
RIM029	18.412	15.686	38.581	0.852	2.096	< 1%	> 15%
HLB356	18.419	15.684	38.658	0.851	2.099	< 1%	> 15%
RIM010	18.409	15.675	38.543	0.851	2.094	< 1%	> 15%
RIM036	18.392	15.656	38.472	0.851	2.092	< 1%	> 15%

Collectively, there are three most distinctive analytical matches in terms of possible ore resources. All these scenarios are historically plausible and can be thus discussed further:

I. The mixing of Mediterranean sources has been thoroughly discussed for the Pb pipes from Pompeii, which had Pb isotope signature close to the samples from this study (Fig. 9; Suppl. Figure 7)^78^. The authors interpreted lead from Pompeii as a mixture of Sardinian, Iberian and Laurion ores; however, as recently pointed out^60^, the possibility of the involvement of the Massif Central ores was initially omitted from the discussion. Considering the original 'mixing scenario', a more satisfying explanation for Pb in the Pompeiian pipes would favour the Cartago Nova deposits with a minor influence from Sardinian ores^60^.
II. The Alpine origin of brass is unsupported because of the lack of clear historical evidence of Roman copper or lead mining in this region. There is a partial Pb isotope overlap with the deposits from the Central Alps (Valais) that may be associated with the 'Sallustian' copper, mentioned by Pliny the Elder, and linked to the Haute Savoie (Suppl. Figure 5, 6), which was discussed in the context of the chemical composition of Lyon altar series AES coinage. This explanation, however, was abandoned because of the inconsistency of the LAS coinage with the Pb isotope ratios of given deposits^71^. A subordinate correlation of the chemical composition and Pb isotope compositions of the 'N' category of samples can be considered for geographical reasons, but this consistency is far from being proven.
III. According to Leblanc's^79^ map, the Pb–Zn mineralisation in the Massif Central is spread from Les Malines to Lyon, where the production of brass is dated from the middle of the first century AD to the beginning of the second century AD^80,81^. The south-eastern part of the Massif Central is rich in various Cu-bearing ore bodies with specific combinations of the trace elements, for example, ophiolites (Ni, Co, Ag), pyrite ores (Ag, Au), Permo-Triassic (As, Pb, Ag), and Hercynian veins (Sb-Ag-Pb) with the Salsigne type mineralisation (As, Bi, Au)^79^. The connection of the polymetallic deposits in the Massif Central^61^ with the Roman lead metallurgy has been suggested earlier^59^. To support this argumentation, six samples in this study that are made of Sn-bronze, Pb-bronze or Ag-rich bronze, i.e. without any cementation process possibly taking place, still have their Pb isotope compositions consistent with the Massif Central ores, and we may thus assume that even Cu was extracted in the same region. A specific mining site, consistent with the Pb isotopic data from this study, cannot be assigned because the available data cover the entire Pb isotope diversity of the Massive Central ore deposits^60^. At present, this dataset appears to bear similarity with Pb isotope values from the Les Malines Pb–Zn deposit^61^. Whether the deposits in the Cevénnes area also served as a Cu source remains uncertain^2^.

### Consideration of possible contamination

A particular methodological risk should be considered when comparing brass artefacts with possible Cu ores because Zn ores, as an essential constituent of the produced brass, may also contain trace amounts of Pb^2,6,82,83^. The inclusion of such Pb may then disturb or obscure the Pb isotope signature of the intrinsic Cu source during the cementation process^84^. There is also the uncertainty on how exactly and how much the cementation medium had impacted the trace element composition, which is crucial for the correct interpretation of the chemical composition of the analysed brass artefacts. So far, it is known that at least Fe and As can enter Cu metal during the cementation process^84^. In Roman Imperial workshops, where a very pure Cu was manufactured due to the advanced refining^13^, the risk of contamination could be exceptionally high. Therefore, it must be acknowledged that the Pb isotope signal from the samples may point to the Pb–Zn source ore instead of the Cu ore [cf.^2^]. Furthermore, the hypothetical contamination during the cementation process could strongly influence the comparison of Roman Cu coins and brass artefacts based on trace elements such as Sb and Ag. These notions, however, require carefully controlled metallurgical experiments. We assume that the cementation process was carried out using Zn in Pb–Zn ore rather than Zn in the form of ZnO, typically developed in furnaces during the pyrotechnological process [cf.^6^]. The Pb contents in brass samples from this study are significantly higher than those in the LAS I coinage^71^, representing at present the purest available copper from the Massif Central.

The possibility of Zn source for Roman brass production was recently further discussed by S. Merkel^77^, who analysed the Zn ores from Dossena in Northern Italy by means of Pb isotope analysis. A slight overlap in Pb isotope compositions of the Dossena Zn ores with the oldest (LT) and the youngest artefacts (R B2) in our sample suite with the tendency towards less radiogenic ^207^Pb/^204^Pb and ^206^Pb/^204^Pb ratios can be observed (Fig. 5a; Suppl. Figure 5, 6). However, the majority of new data from this study are inconsistent with the Zn ores presented in this study. Collectively, we posit that the Massif Central ores still represent the most plausible source of Zn.

### Roman brass production in the Massif Central area

Archaeological evidence for copper mining in the southern Massif Central during the Roman period is still rare; however, two sites in the Cavénes area served as Cu mines during the late first century BC and the early Imperial period^85^. One deposit around Carcassone, exploited during the later Roman Republic (second and first century BC), has also been documented^71,86^. In general, there always is a possibility of missing archaeological evidence of past extraction activities due to medieval and later mining that may have obscured or eradicated the traces of earlier exploitation. Therefore, clear evidence of the Gallic metal supplies is still missing^17^.

The best evidence for the early Roman (i.e. Augustan period) mining in the Massif Central is the Pb isotope analysis of the AES coinage of the so-called Lyon altar series I^71^, supposedly originating in the Cévennes part of the Massif Central. Despite the fact that a part of the Roman brass coin production took place in the Lugdunum (Lyon) mint^71^, similarly to Pb ingots of Santa Maria and partially also to Pb pipes from Pompeii, an Iberian origin was initially expected. However, as the chemical analysis of further Lyon altar series coins (the LAS II collection of the AES coinage) indicates, the Gallic production alone might not have been sufficient for the great demand for Cu during the reign of Augustus, and another Cu source (possibly of the Iberian origin) was thus used for this other series of ases^71^. From the historical perspective, there has been a suggestion that copper used for coins of the LAS I may have been the 'Livian' Gallic copper, mentioned by Pliny the Elder, as one that was quickly depleted^71^.

It should be noted that also other Pb objects have been assumed to originate in the Massif Central, including the artefacts found in Germania^87^. Recently, another assemblage of Roman and Byzantine Cu and Cu-alloy coins *nummi minimi* from the fourth to eighth century AD was found to be consistent with the deposits in the Massif Central, suggesting a long-term mining tradition of local mineral resources^88^. On the other hand, critical notes have also cast some doubt on the Lyon crucibles, pointing out their lack of technical properties^6^. Nevertheless, based on the reasons presented above, we are confident that our data, in fact, indicate the early Roman cementation in the south-eastern part of the Massif Central, and the increasing evidence for the Imperial exploitation of Cu, Zn and Pb in general^59,71^ supports the original interpretation of the Lyon crucibles^80,81^.

The origin of most of the Bohemian brass in the Massif Central, and possibly its fabrication directly in Lyon, is not entirely impossible, as it is in accordance with the recent research, regularly pointing out the Gallic production^2,59,71,87^. The consistency of these deposits with the data from an entirely different cultural tradition may appear surprising at first; however, they only underscore the complexity of the socio-economic networks and the organisation of the metal supplies taking place already in the Early Roman period. Given the presence of the mint in Lugdunum (Lyon), a hypothetical origin of brass for the imperial coinage in the Massif Central appears to be very likely and should be verified by further analyses. The proximity of numerous rich ore deposits to Lugdunum was undoubtedly crucial for its economic importance. These indices could have been underestimated before the publication of chemical data from this area that supported the ancient exploitation of local resources. Furthermore, the consistency of the Pb isotope compositions with samples from the late Iron Age may indicate a long-distance distribution of these mineral resources as soon as around the middle of the first century BC, i.e. the time directly around Caesar's military campaigns in Gaul.

## Conclusions

The majority of samples from this study were made of high-quality brass, arguably of the Roman origin. The Pb isotope data show a clear consistency with ore deposits in the Massif Central, especially with Pb–Zn deposits near Les Malines. In addition, a high degree of homogeneity of the analysed samples in terms of their Pb isotope ratios probably excludes recycling using significantly different resources. Whether the Massif Central connection is provided by Pb contained in the Cu source (metallic or geological), or is a result of the cementation process, cannot be unambiguously distinguished. Given the high purity of the Roman Cu – known from the Cu ingots and Cu-based coinage – with low Pb levels compared to slightly Pb-enriched brass samples, a Pb isotope signal linked to the Pb–Zn ores is more likely. Nevertheless, as indicated by non-brass samples from our assemblage and local provenance of the AES coins of the first altar series from Lyon^71^, even Cu could come from the same territory.

The archaeological cultural groups used to categorise samples appear to have only a moderate significance in the pattern of trace element composition; even the Pb isotope ratios were not influenced significantly compared to categorisation of the samples according to dating. If we accept the possibility that the Pb isotope compositions refer to lead originating in the cementation medium, the variation in trace element patterns may point to various Cu sources, or it may be a result of some further admixtures. Such fact does not contradict a possible different origin of a given artefact suggested by its typological classifications and refers solely to the material used for its fabrication.

An important message provided by the combined chemical and Pb isotope analysis is the inconsistency of brass artefacts with contemporary brass coinage, the Roman *sestertii*. However, this finding requires verification by future analysis of a more varied selection of brass coins using the state-of-the-art analytical methods.

The best level of consistency is found among samples with high Zn content from the phase R B1, i.e. a period around the turn of the Era and the following five decades of the first century AD. Already the artefacts from the late Iron Age do not fall outside the range of Pb isotope ratios of the Massif Central deposits. Therefore, it can be assumed that the brass production might have started in the Massif Central as early as around the middle of the first century BC. The existence of Gallic brass coins from the time of Caesar's military campaigns in Gaul supports this hypothesis^67^. Also, there is evidence of large-scale exploitation of Au in the Massif Central that took place already prior to the Roman conquest^89,90^. It is generally accepted that the Romans benefited from the developed tradition of local Gallic mining^91^. The importance of natural resources in the Massif Central for the expanding Roman Empire is underlined by the intensive Fe production around the Montagne Noire area that became significant in the first century AD. According to archaeometric analyses, local Fe was distributed widely via long-distance trade and served as a vital source of material for the Roman army^92,93^.

The influx of brass to the territories north of the Alps occurred as early as in southern Europe and the Gaul, thus indicating the instant popularity of the new and attractive material. The earliest evidence of brass used in diplomatic contacts with the indigenous populations can be already seen in the late Iron Age. The nature of its distribution mechanisms is hard to evaluate, but in that period, brass was still a rare commodity. Massive-scale and, perhaps more importantly, a regular occurrence is dated no earlier than the Augustan and Tiberian Era. Brass became a ubiquitous yet still highly valued commodity in Germanic society. Its special social status was derived from its distinctive visual qualities and, initially, its exclusive Roman provenance. The level of dependency of the Barbarian society on an external material supply from the Romans appears to be very high. Based on the current scientific evidence, the importance of brass in political relations between the Romans and Barbarians, possibly similar to the role of the silver coins in northern Britain^94^ can be assumed. This material with the connotations of prestige and luxury could serve as an effective medium in determining the quality of relations among different Barbarian groups. The 'value to cost-effectiveness ratio' for the material such as brass seems to have played in the Roman favour. The archaeological evidence from other regions beyond Bohemia suggests that a similar strategy of diplomatic contacts may also apply to other territories where early Roman brass artefacts occur (i.e. Slovakia, Poland, Germany)^95^.

Given the sufficient influx of the Roman brass into the Barbarian territories, the recycling has not affected the geochemical properties attributed to the original Roman *aurichalcum* as much as is observed for materials from the second century AD^66,96^. Only the samples from the earliest phase of the Early Roman period (R A) may have had their Pb isotope ratios influenced by Iberian Cu sources. A specificity of data from the latest phase (R B2) could be explained at this point by exploitation of different deposits in the Massif Central than in the early stages of brass production. Such a hypothesis is also supported by the second century AD brass ingots from the shipwreck of Aléria that share the Pb isotope signature with our samples and is also thought to be produced in the Massif Central. Another explanation brings the recently analysed Zn ores from Northern Italy into consideration as well^77^. Naturally, mixing of various resources in these later stages of Roman brass production is always an important issue for consideration and hopefully will be addressed in future studies on this topic.

The volume of material entering the Germania Magna in the Early Roman period is hard to estimate and represents a research topic on its own. There have been some rough estimations in the work of Becker^97^ for the Barbarian territory of the late Roman Germania, which led to an estimated 2.5 tons of material just for brooches. Given the larger dimensions of the Early Roman brooches compared to Late Roman types, plus the overall abundance of the metallic goods in the Early Roman graves, the quantity of consumed material must have been probably higher than that estimated.

## Methods

Selected artefacts were drilled to the metal core to avoid the corrosion layers and collect the minimum sufficient amount of material for the chemical and Pb isotope analyses. Due to the small sizes of the artefacts and the high corrosion stage of some, the sample weight varied between 0.01 and 0.05 g. Because of the sample preparation methodology, As contents were not determined.

Samples of drilled-out bronze/brass materials were carefully weighed into pre-cleaned Savillex beakers, dissolved in a mixture of 6 M HCl–7 M HNO_3_ (3:1 v/v) with several drops of 23 M HF and placed on a hotplate for 24 h at 50 °C. For the measurements of element abundances, freshly prepared solutions were dried down and re-dissolved in 2% HNO_3_. The abundances of selected elements were determined using an Agilent 7900x inductively coupled plasma mass spectrometer (ICP-MS), housed at the Czech Geological Survey.

The chemical procedures for Pb isolation and purification employed two chromatographic columns. The first step was modified from Pin et al.^98^ and used pre-cleaned and pre-conditioned Sr.Spec resin (50–100 mesh; Triskem, France) packed in 0.2 mL columns. Samples were dried down and re-dissolved in 2 M HCl. Lead was eluted with 6 M HCl. The second step employed anion-exchange resin BioRad AG 1 × 8 (100–200 mesh) combined with HCl and HBr as elution media, following the methodology outlined in Romer et al.^99^. The eluted Pb fraction was then dried down and repeatedly re-dissolved with 50 μl 14 M HNO_3_ to remove any residual organic material.

Prior to Pb isotope measurements, the dried Pb fractions were re-dissolved in 1 mL 2% HNO_3_ and doped with Tl solution (NIST SRM 997; ^205^Tl/^203^Tl = 2.3871). Lead isotope compositions were determined using a Neptune multi-collector ICPMS (ThermoFisher) coupled to an Aridus 2 desolvating unit (Cetac), housed at the Czech Geological Survey, in static mode. Sample analysis followed a conventional standard–sample–standard bracketing protocol in which the SRM-981 reference material solution was run after every unknown sample. Potential ^204^Hg isobaric interference on ^204^Pb was monitored at mass ^202^Hg and corrected by assuming natural Hg isotope ratios (^202^Hg/^204^Hg = 4.35). Correction of the measured Pb isotope ratios for mass discrimination utilised a generalised power law and natural isotope composition of Tl^100^. The results were then normalised off-line to the certified values for SRM 981, and the combined statistics for three measurements of each unknown sample were calculated. Data represent the uncertainty-weighted mean of three replicate measurements. Repeat measurements of NBS 981 yielded mean ^206^Pb/^204^Pb, ^207^Pb/^204^Pb and ^208^Pb/^204^Pb ratios of 16.942 ± 0.003, 15.4998 ± 0.0030 and 36.725 ± 0.007 (2SEM, n = 66), respectively.

Samples dated into the La Tène period and comparative Iron Age samples were processed with a slightly different methodology detailed elsewhere^21^.

For consistency in the data evaluation, the ore deposits data were prepared for the comparative analysis by removing multivariate outliers (i.e. those that would significantly affect the quality of the analysis) detected using the Mahalanobis distance^76^. This step is a prerequisite to fitting linear models to the data or using any other method to visualise the trends. By outliers, we understand data points with extreme values regarding the shape of the whole data distribution in a multidimensional setting defined by lead isotopic ratios (^206^Pb/^204^Pb, ^207^Pb/^204^Pb, ^208^Pb/^204^Pb, ^207^Pb/^206^Pb and ^208^Pb/^206^Pb, respectively).

## Supplementary Information

Supplementary Information 1.

Supplementary Information 2.

Supplementary Information 3.
