# Supplementary material for: Archaeometric perspective on the emergence of brass north of the Alps around the turn of the Era
Source: Sci Rep. 2022 Jan 10;12:374. doi: 10.1038/s41598-021-04044-7 (PMC8748637; doi:10.1038/s41598-021-04044-7)
Supplement: Supplementary file 1 — Supplementary Information 1. [file 41598_2021_4044_MOESM1_ESM.pdf]

Archaeometric perspective on the emergence of brass north of the Alps around the turn of the Era

Daniel Bursák[a]*, Alžběta Danielisová[a], Tomáš Magna[b], Petr Pajdla[c], Jitka Míková[b], Zuzana Rodovská[b], Ladislav Strnad[d], Jakub Trubač[d]

a Institute of Archaeology of the CAS Prague, v.v.i., Letenská 4, CZ-118 01 Prague 1, Czech Republic

b Czech Geological Survey, Klárov 3, CZ-118 21 Prague 1, Czech Republic

c Department of Archaeology and Museology, Faculty of Arts, Masaryk University, Joštova 220/13, 662 43, Brno, Czech Republic

d Institute of Geochemistry, Mineralogy and Mineral Resources, Faculty of Science, Charles University, Albertov 6, CZ-128 43 Prague 2, Czech Republic

* corresponding author (bursak@arup.cas.cz)

Supplementary information figures and table:

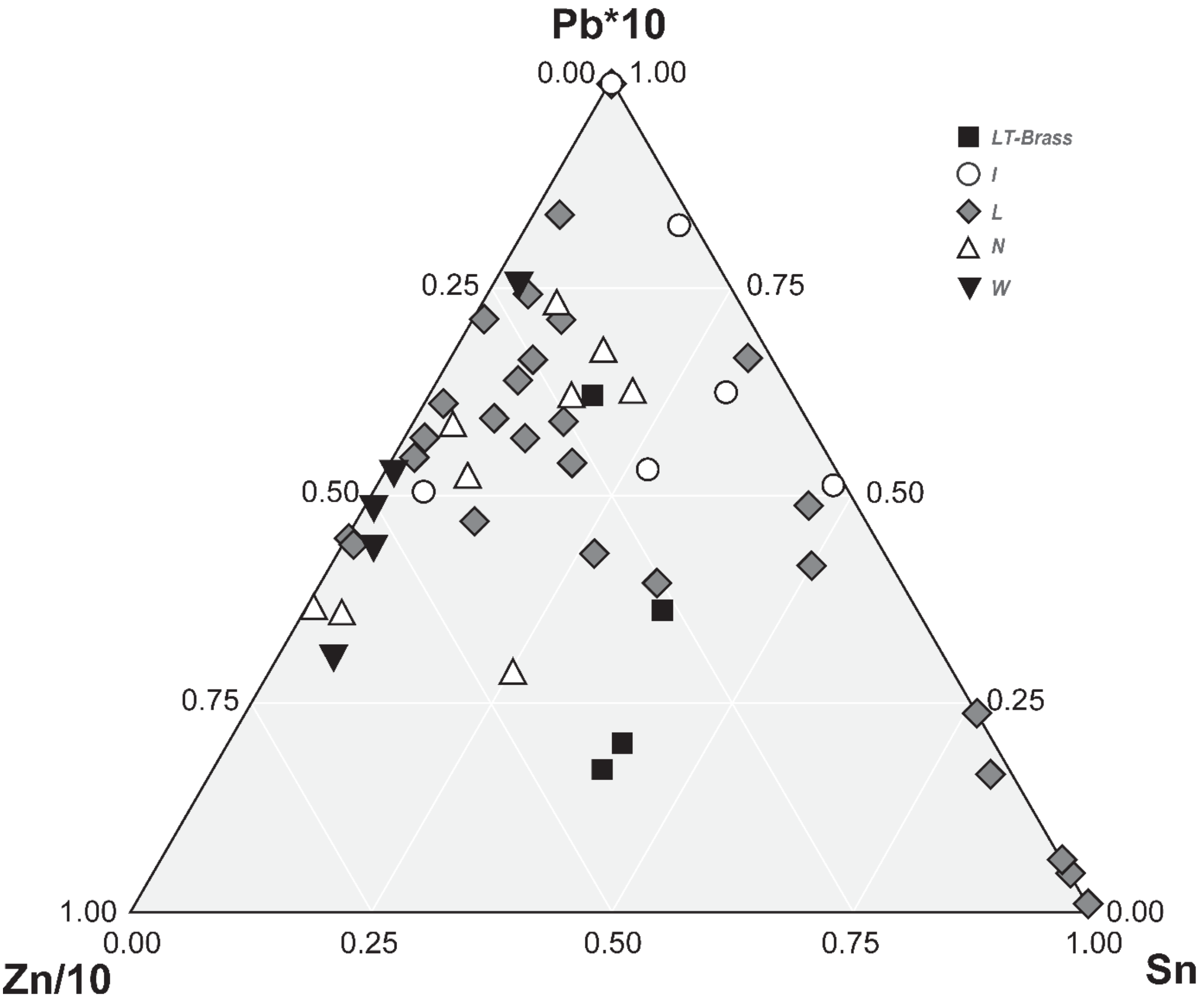

Suppl. Fig. 1: Ternary Pb–Zn–Sn plot for the Bohemian brass samples categorised according to the cultural groups. Zinc content is divided by a factor of ten, whereas the Pb content is multiplied by a factor of ten.

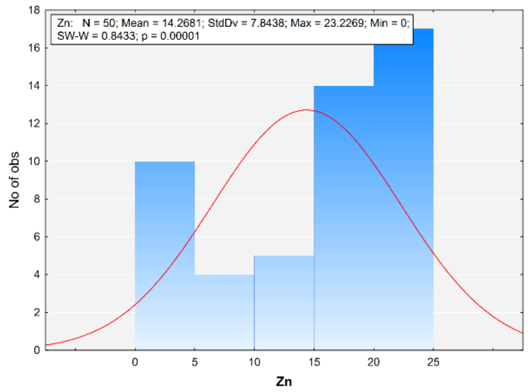

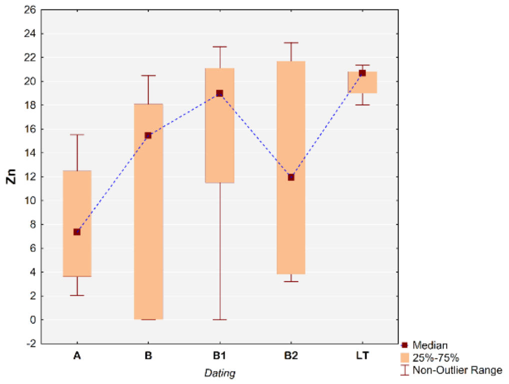

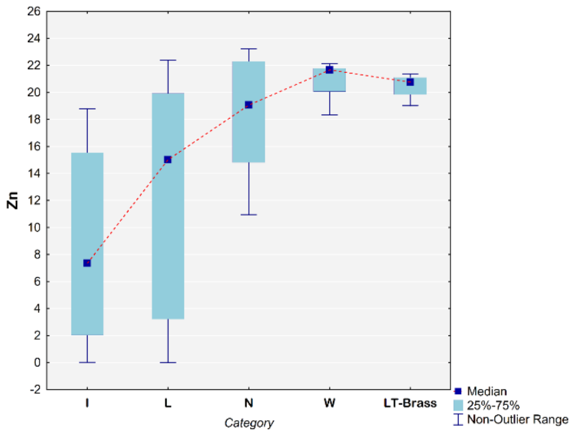

Suppl. Fig. 2: The Zn content in the Bohemian brass samples plotted as A: histogram; B: boxplot according to dating; C: boxplot according to the cultural groups.

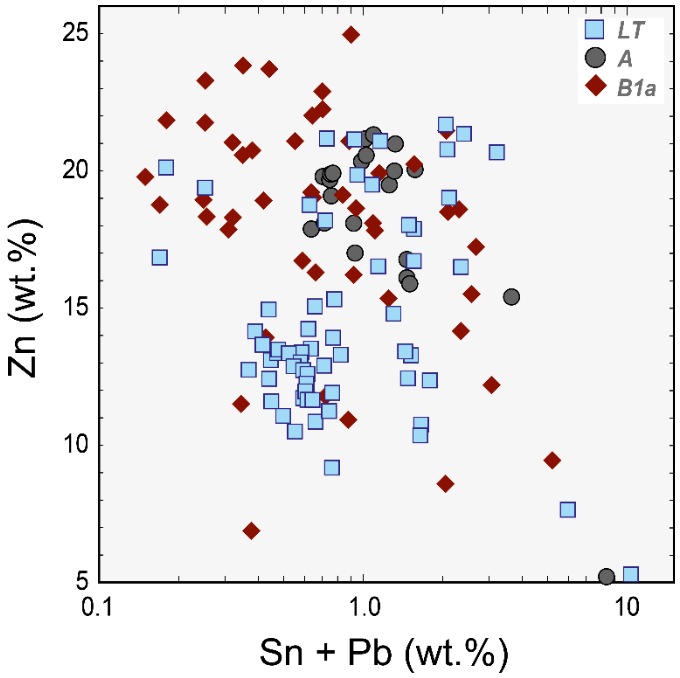

Suppl. Fig. 3: Plot of Zn verus Sn + Pb contents (in wt. %) of the earliest brass objects from the 1st century BC categorised according to dating. Sources: [1–3] + this study.

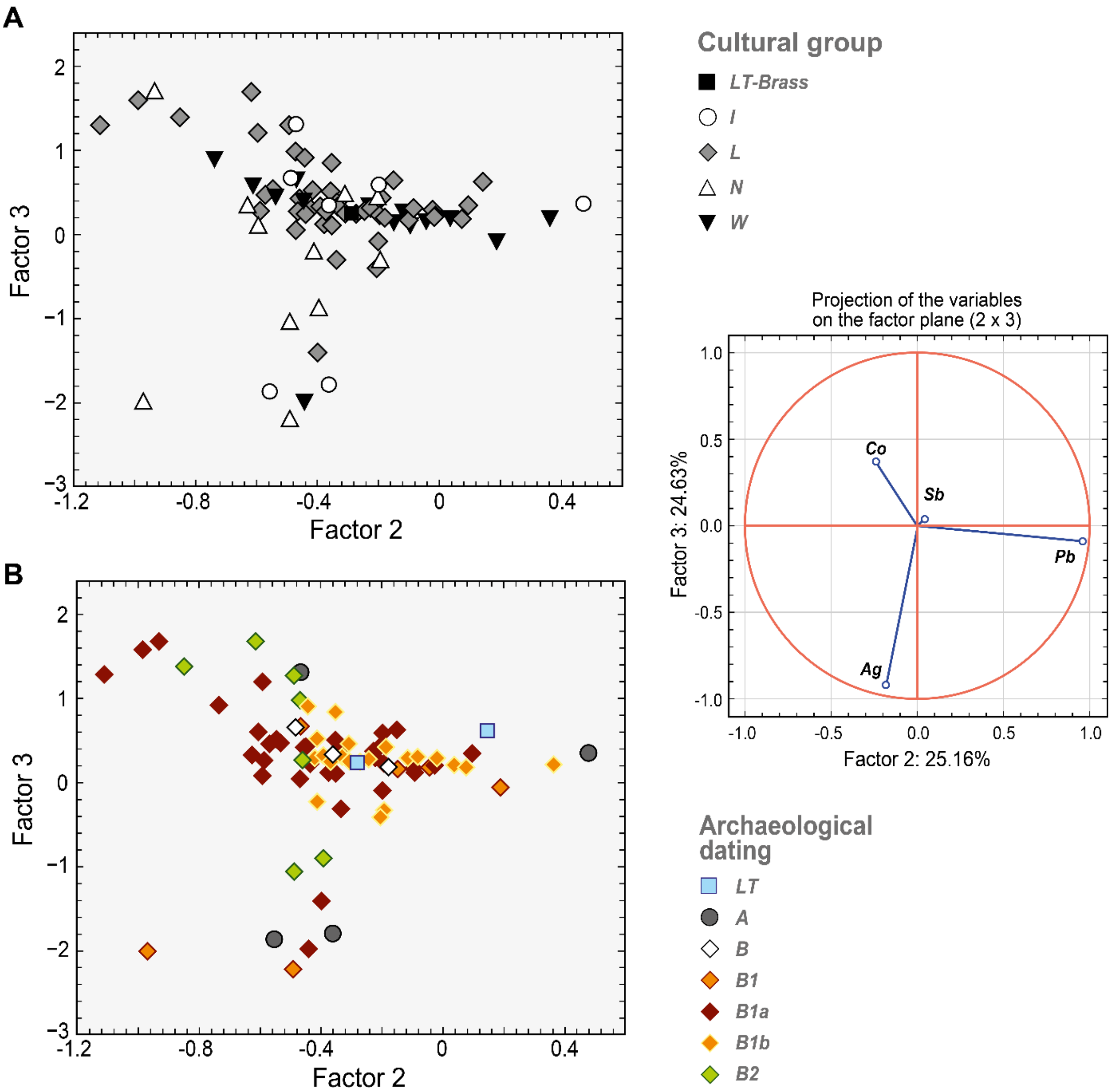

Suppl. 4: Bi-plot with results of principal component analysis based on minor/trace elements (Pb, Co, Sb, Ag) for selected brass objects from Bohemia with the comparative datasets. Factor 1 versus Factor 2 categorised according to the cultural groups (A) and dating (B). Sources: [1–4] +this study. The inset panel shows variables factor map.

(A) Euclidean distance

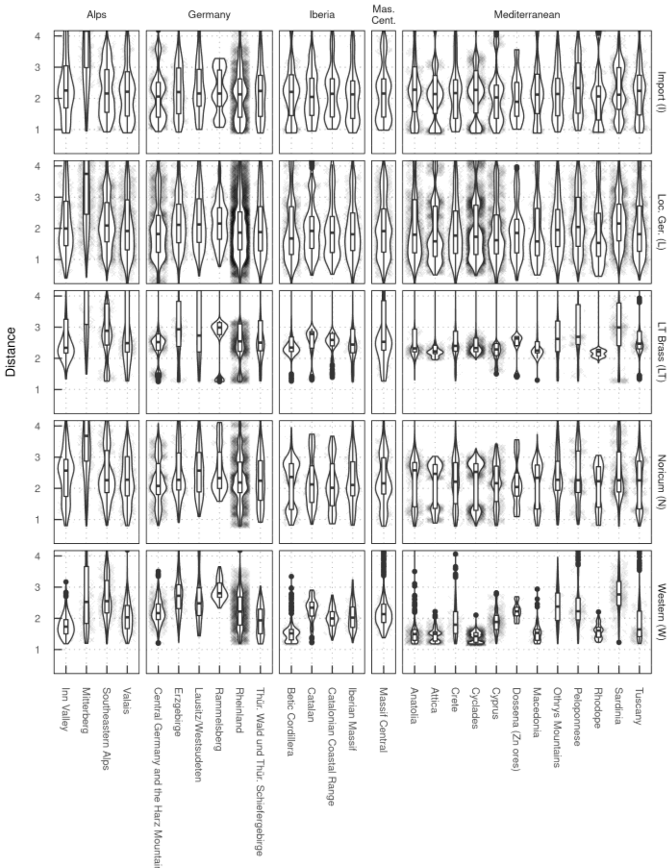

(B) Mahalanobis distance

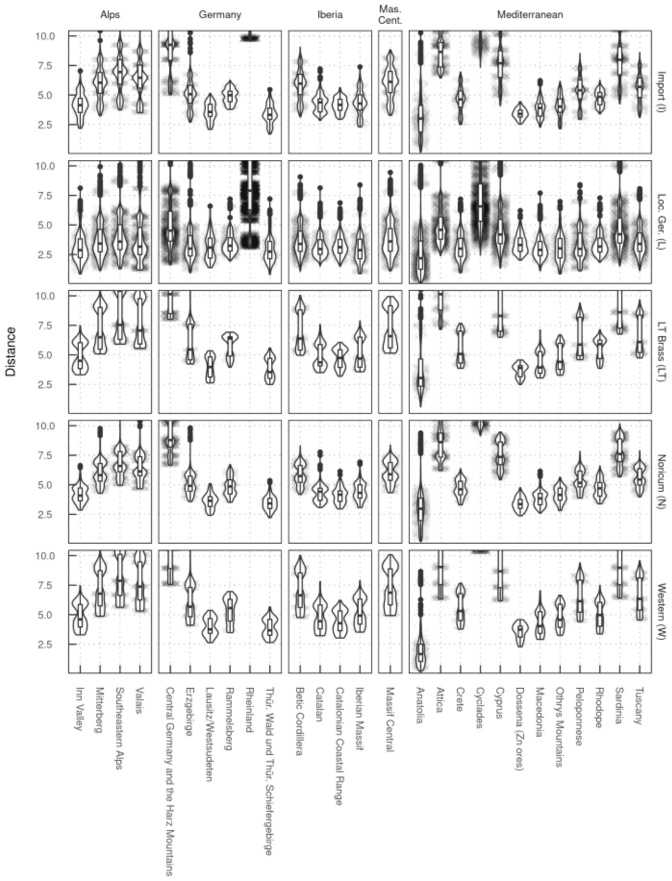

Suppl. Fig. 5: Euclidean (ED) and Mahalanobis (MD) distances of lead isotope ratios of various ore sources from Europe and the Bohemian brass objects categorised according to the cultural groups. Sources: see [5–15].

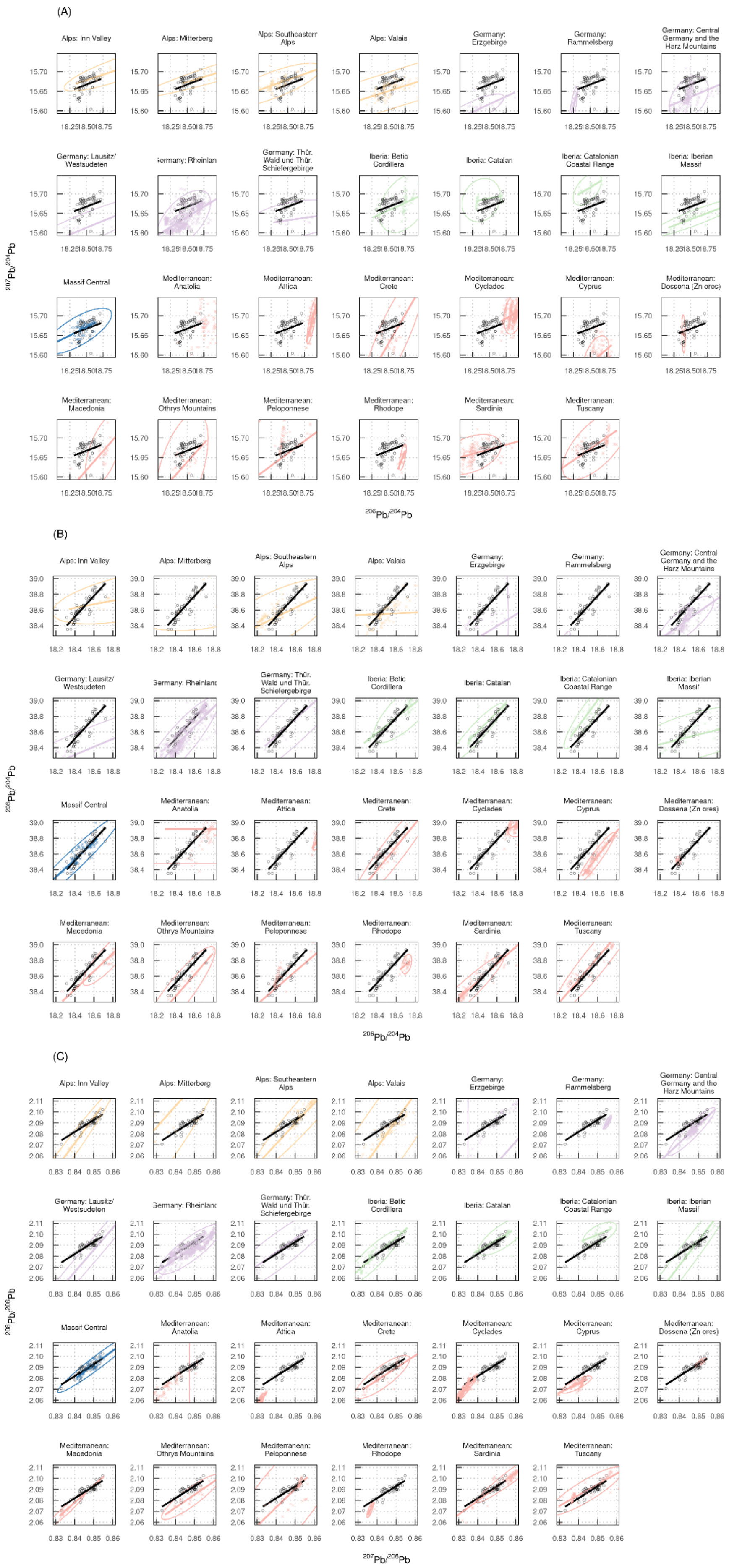
(A)
Alps: Inn Valley
Alps: Mitterberg
Alps: Southeastern Alps
Alps: Valais
Germany: Erzgebirge
Germany: Rammelsberg
Germany: Central Germany and the Harz Mountains
Germany: Lausitz/ Westsudeten
Germany: Rheinland
Germany: Thür. Wald und Thür. Schiefergebirge
Iberia: Betic Cordillera
Iberia: Catalan
Iberia: Catalonian Coastal Range
Iberia: Iberian Massif
Massif Central
Mediterranean: Anatolia
Mediterranean: Attica
Mediterranean: Crete
Mediterranean: Cyclades
Mediterranean: Cyprus
Mediterranean: Dossena (Zn ores)
Mediterranean: Macedonia
Mediterranean: Othrys Mountains
Mediterranean: Peloponnese
Mediterranean: Rhodope
Mediterranean: Sardinia
Mediterranean: Tuscany
$^{207}Pb/^{204}Pb$
$^{206}Pb/^{204}Pb$
(B)
$^{208}Pb/^{204}Pb$
$^{206}Pb/^{204}Pb$
(C)
$^{208}Pb/^{206}Pb$
$^{207}Pb/^{206}Pb$

Suppl. Fig. 6: Comparison of slopes and linear models of the lead isotope systematics of possible ore sources and the Bohemian brass objects. Sources: see [5–15].

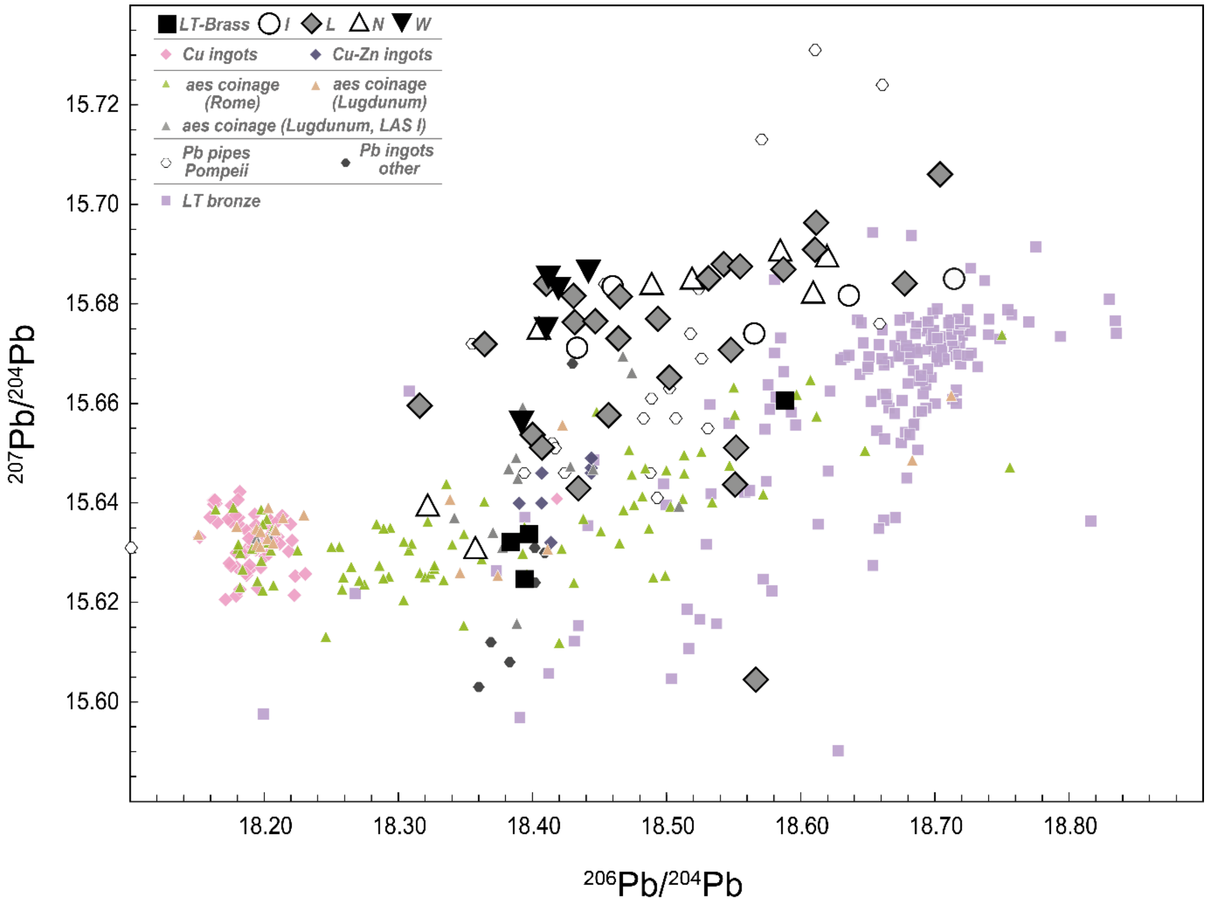

Suppl. Fig. 7: Overview of lead isotope systematics of various sets of artefacts from the Early Roman period and the Bohemian brass objects categorised according to cultural groups. Sources: see description in chapter 4.2 + this study.

Sample	Collection	Artefact inv. no.	Artefact description	Site	Dating
RIM 001	Collection of the National Museum Prague, Czech Republic	H1-469906	pin with small sphere	Mlékojedy, Central Bohemia	1st century AD
RIM 004	Collection of the National Museum Prague, Czech Republic	H1-469831	pin with profilated top	Mlékojedy, Central Bohemia	1st century AD
RIM 014	Collection of the National Museum Prague, Czech Republic	H1-469899	pin with profilated top	Mlékojedy, Central Bohemia	1st century AD

RIM 017	Collection of the National Museum Prague, Czech Republic	H1-469968	brooch, type Aucissa	Mlékojedy, Central Bohemia	1st century BC - early 1st century AD
RIM 023	Collection of the National Museum Prague, Czech Republic	H1-469824	brooch, type Almgren 2a	Mlékojedy, Central Bohemia	1st century BC - early 1st century AD
RIM 025	Collection of the National Museum Prague, Czech Republic	H1-469839	brooch, spoon type	Mlékojedy, Central Bohemia	1st century BC
RIM 026	Collection of the National Museum Prague, Czech Republic	H1-469913	pin with small sphere	Mlékojedy, Central Bohemia	1st century AD
RIM 030	Collection of the National Museum Prague, Czech Republic	H1-469846	brooch, type Almgren 18	Mlékojedy, Central Bohemia	1st half of the 1st century AD
RIM 032	Collection of the National Museum Prague, Czech Republic	H1-469865	pin	Mlékojedy, Central Bohemia	1st half of the 1st century AD
RIM 035	Collection of the National Museum Prague, Czech Republic	H1-469859	pin with small sphere	Mlékojedy, Central Bohemia	1st half of the 1st century AD

Suppl. Table 1: Further information of artefacts from the collection of National Museum Prague, Czech republic.

Bibliography:

1. Istenič, J. & Šmit, Ž. The beginning of the use of brass in Europe with particular reference to the south-eastern Alpine region. in *Metals and Mines: Studies in Archaeometallurgy* (eds. la Niece, S., Hook, D. & Craddock, P. T.) 140–147 (London Archetype Publications / British Museum, 2007).

2. Istenič, J. Roman military equipment and the beginnings of the Roman use of brass in Europe. in *Waffen in Aktion. Akten 6. International Roman Military Equipment Conference, Xanten 13.-16. 6. 2007. Xantener Berichte 16* (eds. Busch, A. W. & Schalles, H. J.) 237–242 (Philipp von Zabern, 2009).

3. Riederer, J. Metallanalysen römischer Fibeln aus Kempten. in *Fiblen und Bronzegeвe von Kempten-Cambodunum. Cambodunumforschungen 5. Materialhefte zur Bayerischen Vorgeschichte Reihe A* (eds. Schleiermacher, M. & Flügel, Ch.) 45–52 (1993).

4. Frána, J. Metallanalysen der germanischen und römischen Gegenstände aus Dobřichov-Pičhora. in *Dobřichov-Pičhora. Ein Gräberfeld der älteren römischen Kaiserzeit in Böhmen* (ed. Droberajar, E.) 185–194 (1999).

5. Hanel, N. & Bode, M. Messingbarren aus einem römischen Schiffswrack bei Aléria (Korsika). in *From Bright Ores to Shiny Metals. Festschrift for Andreas Hauptmann on the Occasion of 40 Years Research in Archaeometallury and Archaeometry. Montanhistorische Zeitschrift. Der Anschnitt Beiheft 29.* (eds. Körlin, G., Prange, M., Stöllner, Th. & Yalcin, Ü.) 167–181 (2016).

6. Bode, M., Rothenhöfer, P. & Batanero, D. G. Lost in the south: A Roman copper ingot form the area of Tarragona in the Baetica. *Revista Onoba* **6**, 243–248 (2018).

7. Danielisová, A., Strnad, L. & Mihaljevič, M. Circulation Patterns of Copper-Based Alloys in the Late Iron Age Oppidum of Třísov in Central Europe. *Metalla* **24**, 5–18 (2018).

8. Danielisová, A. *et al.* Claiming the land or protecting the goods? The Duchcov hoard in Bohemia as a proxy for 'Celtic migrations' in Europe in the 4th century BCE. *Journal of Archaeological Science* **127**, (2021).

9. Danielisová, A. *et al.* Rituals, hoards and travellers? Archaeometry of the iron age bronze wheel amulets. *Interdisciplinaria Archaeologica* **11**, (2020).

10. Trincherini, P. R., Barbero, P., Quarati, P., Domergue, C. & Long, L. Where do the lead ingots of the Saintes-Maries-de-la-Mer wreck come from? Archaeology compared with physics. *Archaeometry* **43**, (2001).

11. Artioli, G., Canovaro, C., Nimis, P. & Angelini, I. LIA of Prehistoric Metals in the Central Mediterranean Area: A Review. *Archaeometry* **62**, 53–85 (2020).

12. Sinclair, A. J., Macquar, J. C. & Rouvier, H. Re-evaluation of lead isotopic data, southern Massif Central, France. *Mineralium Deposita* **28**, 122–128 (1993).

13. Merkel, S. W. Calamine of the Bergamasque Alps as a possible source of zinc for Roman brass: Theoretical considerations and preliminary results. *Periodico di Mineralogia* **90**, 247–259 (2021).

14. Stos-Gale, Z. A., Maliotis, G., Gale, N. H. & Annetts, N. Lead isotope characteristics of the Cyprus copper ore deposits applied to provenance studies of copper oxhide ingots. *Archaeometry* **39**, 83–123 (1997).

15. Veron, A., Leroux, G., Poirier, A. & Baque, D. Origin of copper used in bronze artifacts from Middle Bronze Age burials in Sidon: a synthesis from lead isotope imprint and chemical analyses. *Archeaology & History in the Lebanon* 34–78 (2011).
