# Supplementary figures and images for: Archaeometric perspective on the emergence of brass north of the Alps around the turn of the Era
Source: Sci Rep. 2022 Jan 10;12:374. doi: 10.1038/s41598-021-04044-7 (PMC8748637; doi:10.1038/s41598-021-04044-7)

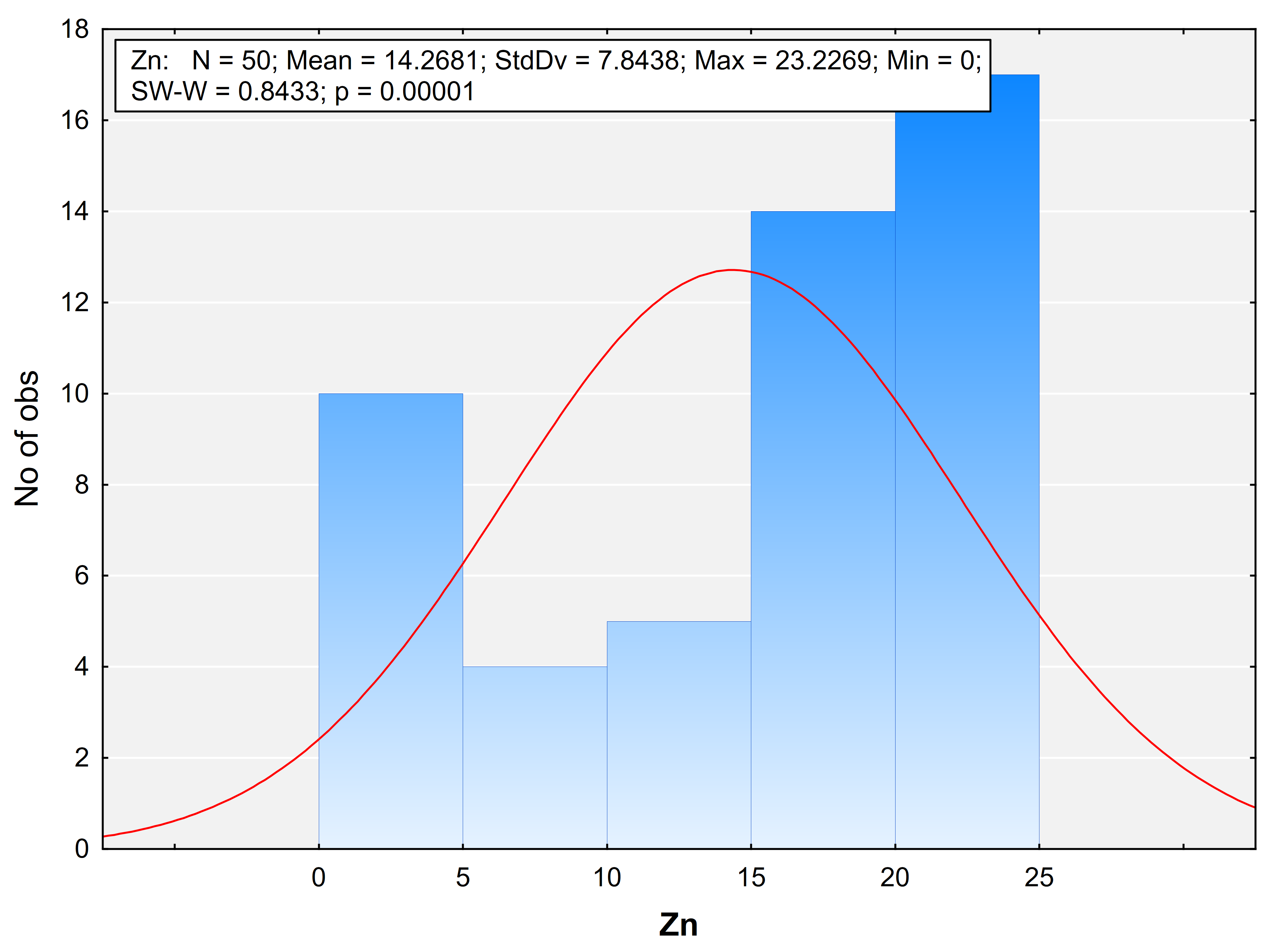

Supplement: Supplementary file 2 — Supplementary Information 2. [file 41598_2021_4044_MOESM2_ESM.zip › Bursaketal_SupplFig2a.tif]

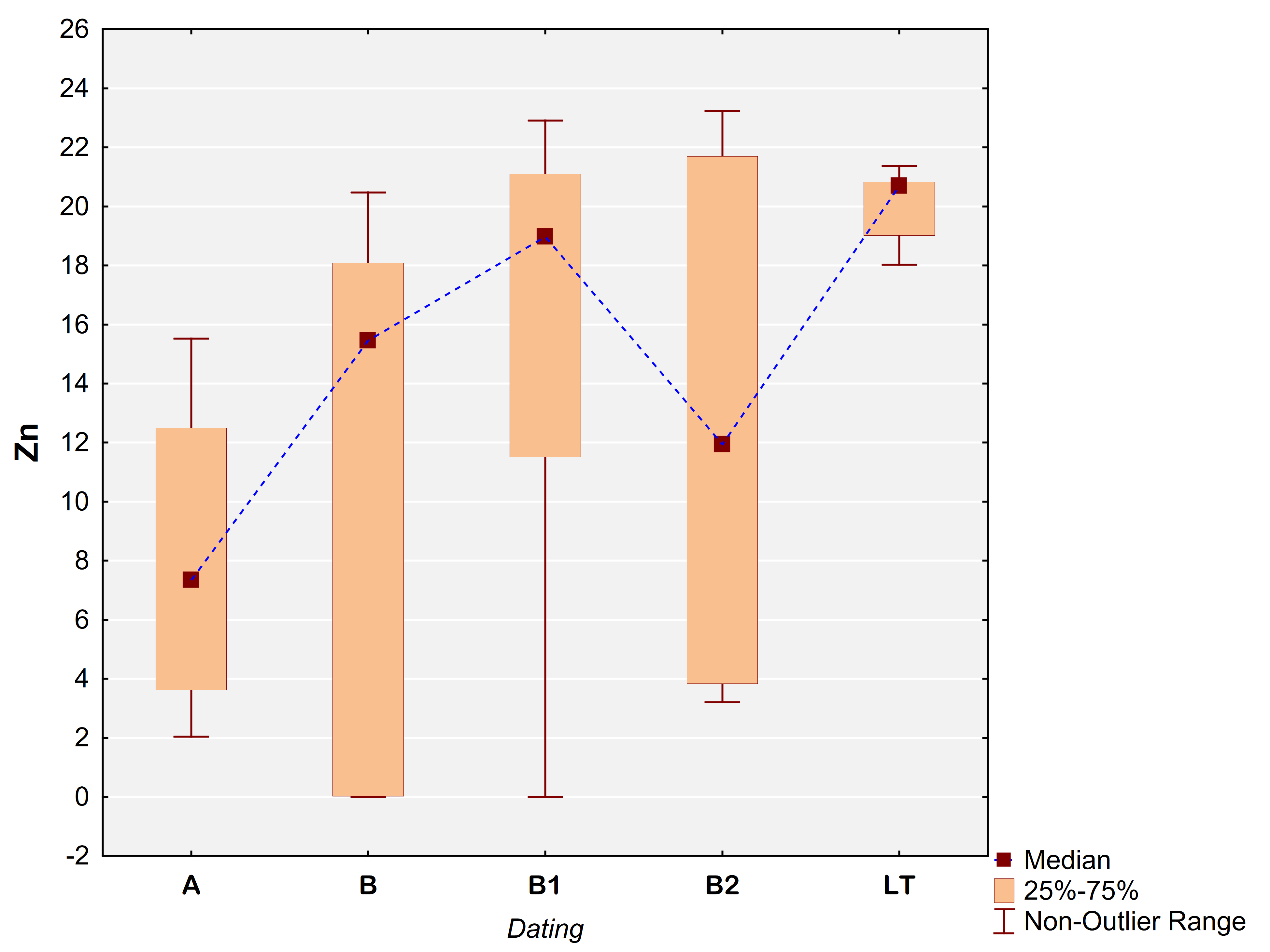

Supplement: Supplementary file 2 — Supplementary Information 2. [file 41598_2021_4044_MOESM2_ESM.zip › Bursaketal_SupplFig2b.tif]

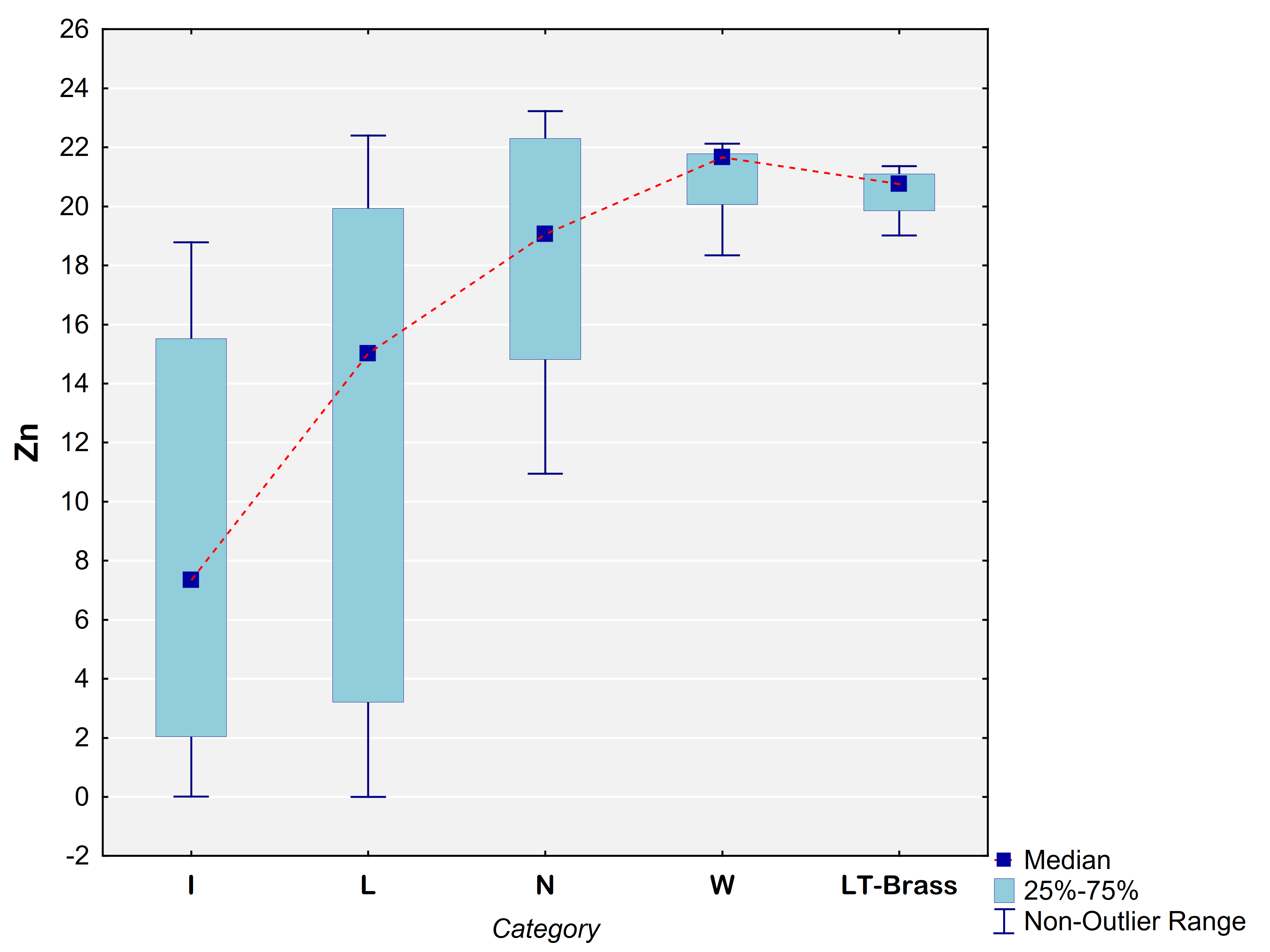

Supplement: Supplementary file 2 — Supplementary Information 2. [file 41598_2021_4044_MOESM2_ESM.zip › Bursaketal_SupplFig2c.tif]

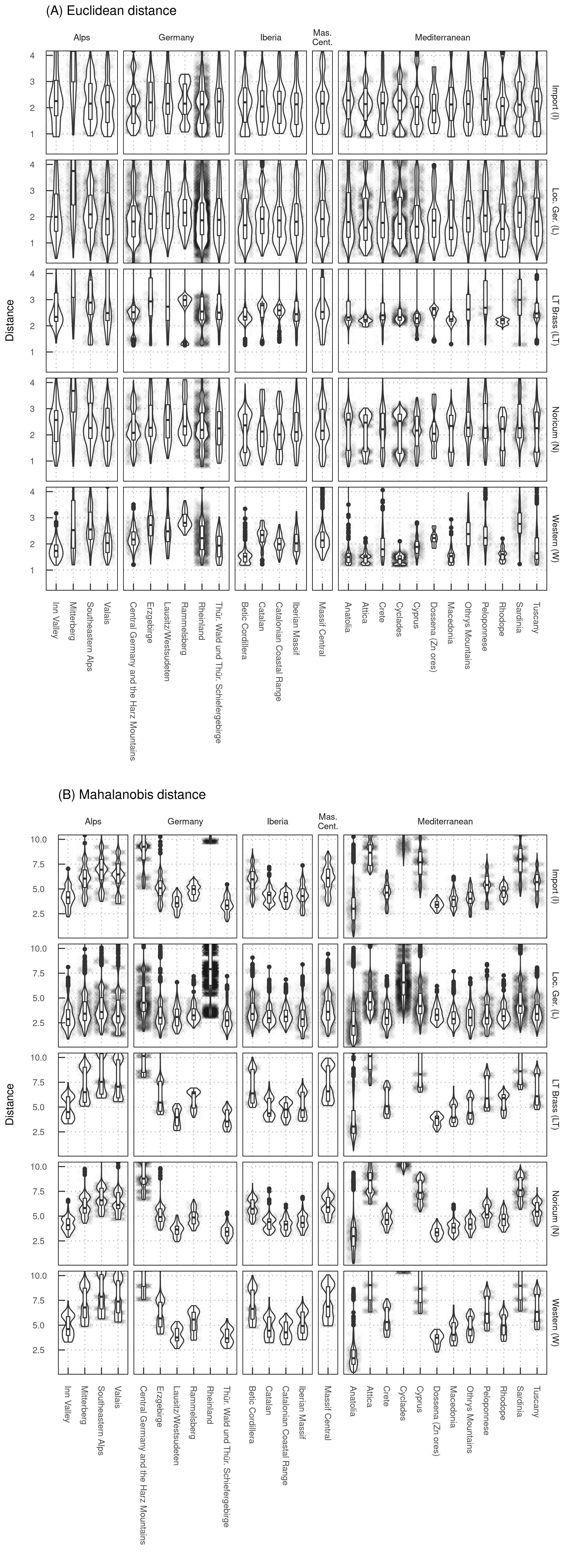

Supplement: Supplementary file 2 — Supplementary Information 2. [file 41598_2021_4044_MOESM2_ESM.zip › Bursaketal_SupplFig5.png]

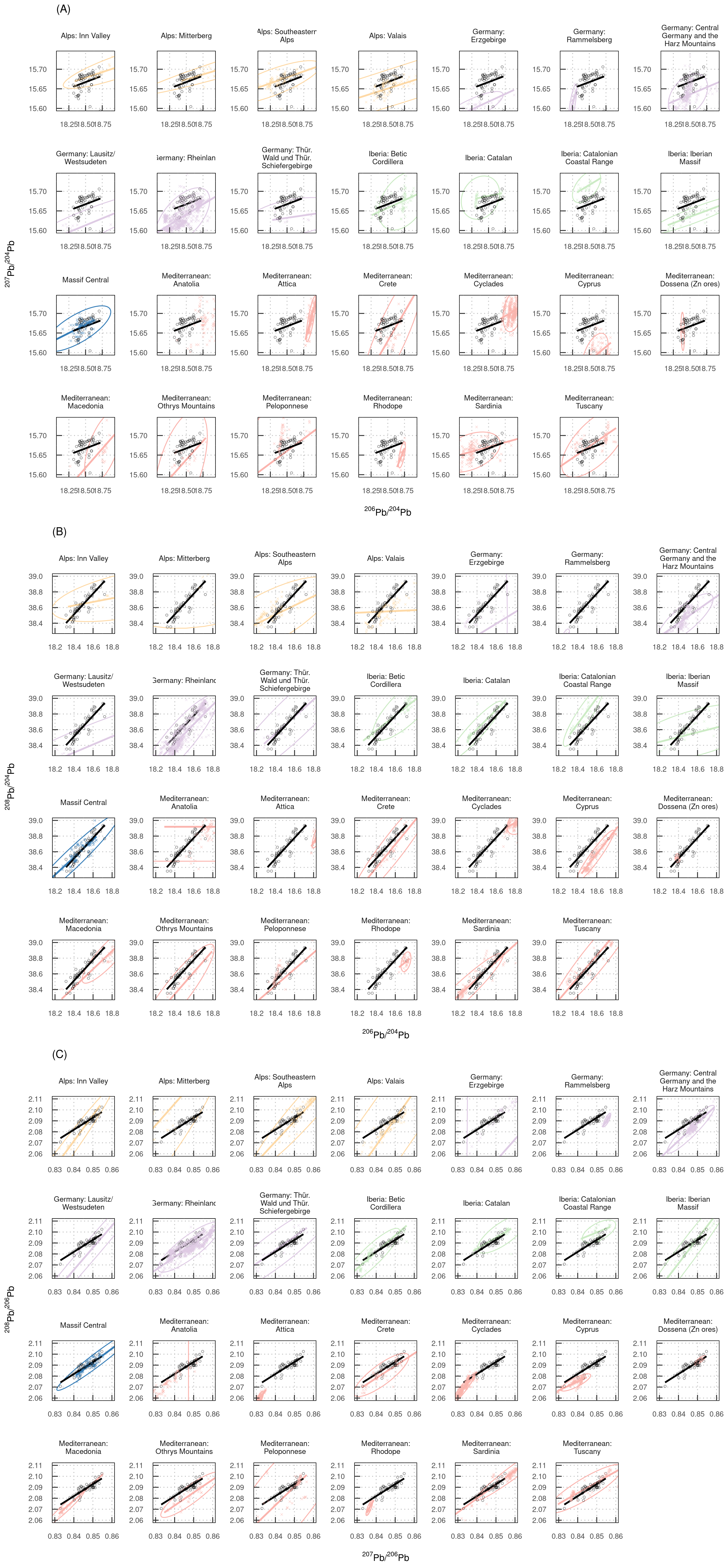

Supplement: Supplementary file 2 — Supplementary Information 2. [file 41598_2021_4044_MOESM2_ESM.zip › Bursaketal_SupplFig6.png]
